# Unraveling the FGFR–RNA splicing axis: Mechanisms, oncogenic crosstalks and innovations for therapeutic purpose

**DOI:** 10.1016/j.apsb.2025.11.031

**Published:** 2025-12-01

**Authors:** Xuquan Xian, Ruyi Gong, Shunzi Rong, Zhihao Zhang, Fengtong Jia, Lin Li, Zhengguo Chen, Beatrice Eymin, Tao Jia

**Affiliations:** a National Health Commission (NHC) Key Laboratory of Nuclear Technology Medical Transformation, Sichuan Provincial Engineering Research Center of Nuclear Medical Equipment Translation and Application, Sichuan Clinical Research Center for Radiation and Therapy, Mianyang Central Hospital, Mianyang 621000, China; b Key Laboratory of Drug-Targeting and Drug Delivery System of the Education Ministry and Sichuan Province, Sichuan Engineering Laboratory for Plant-Sourced Drug and Sichuan Research Center for Drug Precision Industrial Technology, West China School of Pharmacy, Sichuan University, Chengdu 610041, China; c Department of Nuclear Medicine, Mianyang Central Hospital, Mianyang 621000, China; d University Grenoble Alpes, INSERM U1209, CNRS UMR 5309, Team RNA Splicing, Cell Signaling and Response to Therapies, Institute For Advanced Biosciences, Grenoble 38000, France

**Keywords:** FGF–FGFR, RNA splicing, Tumorigenesis, Isoform diversity, Therapeutic resistance, Biomarker discovery, Radiotheranostics, Precision oncology

## Abstract

Fibroblast growth factor receptor (FGFR) signaling is a pivotal regulator of tumor progression, driving cell proliferation, survival, metastasis, and therapeutic resistance across diverse cancer types. RNA alternative splicing profoundly shapes FGFR isoform diversity, endowing tumors with heterogeneity and adaptability to targeted interventions. While significant progress has been made in identifying splicing regulators that govern FGFR pre-mRNA processing, the extracellular cues influencing this process and the reciprocal impact of FGFR signaling pathway on global splicing networks remain underexplored. This review provides a comprehensive overview of the bidirectional interplay linking FGFR signaling and RNA splicing in cancer. Mechanistically, we first detail how *FGFR* mutations, epigenetic modifications, and crosstalks with oncogenic pathways reprogram splicing to generate tumor-specific FGFR splice variants. We then systematically classify distinct FGFR isoforms and delineate how they contribute to main cancer hallmarks, underscoring the central role of the FGFR–splicing axis in driving tumor plasticity, heterogeneity and adaptive progression. Conversely, we also examine how FGFR signaling modulates RNA splicing programs beyond FGFR itself, reshaping global splicing events that contribute to tumorigenesis, an emerging and still largely unexplored area of cancer biology. From therapeutic perspective, we highlight emerging strategies targeting the axis. Notably, FGFR splicing isoform-directed radiopharmaceuticals hold great promise for patient stratification and biomarker-directed theranostics, providing a precise approach to identify aggressive tumors and guide tailored interventions. As well, complementary approaches, including CRISPR/Cas9-based splicing modulation and long non-coding RNAs-targeted therapies, further expand the toolbox for isoform-specific intervention. Moreover, integrating splicing modulators with FGFR TKIs may overcome drug resistance. Understanding the intricate interplay between FGFR signaling and RNA splicing will not only advance biomarker-guided therapeutic development but also provide a novel framework to counteract tumor adaptability, ultimately improving outcomes in FGFR-driven malignancies.

## Graphical abstract

Mechanistic studies uncover the interplay between FGFR signaling, RNA splicing, and tumor progression. Derived therapeutic strategies extend beyond conventional inhibitors, highlighting isoform-specific FGFR modulators, splicing-targeted combination therapies, and emerging radiotheranostic approaches.

**Figure:**
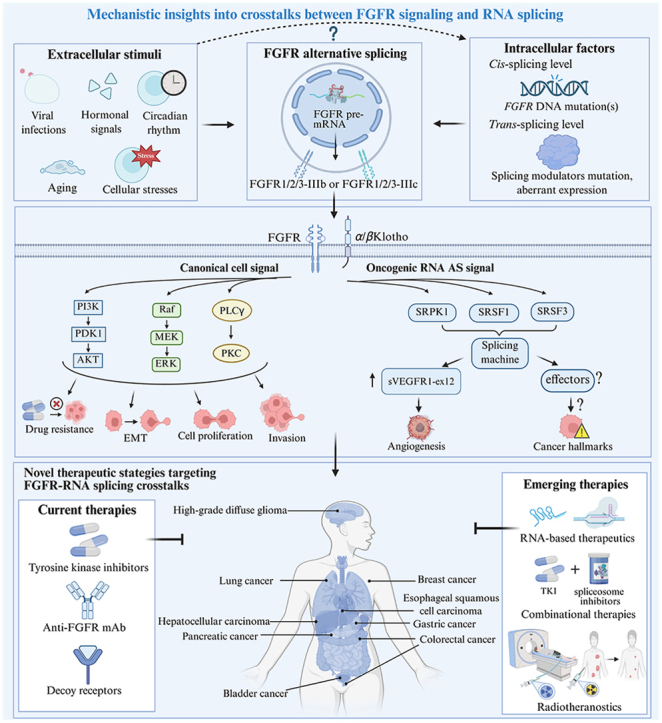

## 1 Introduction

Fibroblast growth factor receptors (FGFRs) belong to the family of receptor tyrosine kinases (RTKs) that consist of an extracellular ligand-binding domain and an intracellular tyrosine-kinase domain. A total of 22 distinct fibroblast growth factor (FGF) ligands are known to interact with and activate the four FGFRs^1^. Upon FGFs binding, FGFRs dimerize and undergo autophosphorylation, recruiting downstream signaling proteins that activate multiple pathways involved in cell proliferation, differentiation, survival, migration, and angiogenesis^2^.

Dysregulation of the FGFR axis through gene amplification, mutations, fusions, chromosomal translocations, or aberrant splicing has been extensively implicated in oncogenesis^3^. Additionally, abnormal FGF expression and disrupted autocrine/paracrine signaling in tumor and stromal cells have been shown to drive carcinogenesis^4^. Moreover, overexpression of key FGFR-associated signaling nodes, such as FGFR substrate 2 (FRS2), amplifies mitogen-activated protein kinase-extracellular signal-regulated kinase signaling, thereby promoting tumor survival^5^. Recent studies have underscored the diverse oncogenic functions of FGFRs in cancer. These include nuclear FGFR1-regulated gene transcription to promote antiestrogen resistance^6^, the FGFR2–nuclear factor-*κ*B axis driving metabolic reprogramming^7^, and FGFR3 phosphorylation-dependent regulation of purine metabolism, S-phase progression, and tumorigenesis^8^. These findings underscore that the diversity of FGF–FGFR interactions surpasses that of other receptor tyrosine kinases (RTKs). Targeting the FGF–FGFR axis, mainly through the use of FGFR–tyrosine kinase inhibitors (FGFR-TKIs) or anti-FGFR monoclonal antibodies, remains a key focus to treat cancer, as highlighted in recent reviews^9^^,^^10^. However, limitations exist as most of the patients exhibit primary or acquire resistance to these treatments. Therefore, elucidating the molecular mechanisms by which FGFR signaling drives tumorigenesis is essential to refine patient selection, enhance the efficacy of FGFR-targeted therapies, and guide the development of novel therapeutic strategies.

RNA splicing is a fundamental process that notably expands the coding capacity of the genome. The spliceosome, a highly dynamic complex, accurately removes introns and joins exons to produce mature mRNAs. Alternative splicing (AS) enables a single gene to generate multiple mRNA isoforms^11^. However, disruptions in splicing caused by either somatic mutations or dysregulated expression of RNA-binding proteins can lead to aberrant splice variants that drive tumorigenesis^12^. Given its central role in cancer progression, targeting the spliceosome machinery has emerged as a promising therapeutic strategy in oncology, as discussed in our recent review^13^.

AS plays a critical role in FGFR-driven pathologies, including oncogenesis. While the effects of various FGFR splice variants have been widely studied in this context, the upstream signaling pathways that control aberrant FGFR alternative splicing, as well as the role of FGFR signaling itself in reprogramming RNA splicing to drive tumorigenesis and drug resistance, remain poorly understood. To bridge this knowledge gap, we provide, to the best of our knowledge, the first comprehensive review of the bidirectional interplay between FGFR signaling and RNA splicing. Hence, we mechanistically examine how alternative splicing governs FGFR isoform diversity to impact the main cancer hallmarks, and how FGFR activation, in turn, reshapes global splicing programs to drive oncogenesis and therapeutic resistance.

At the therapeutic level, we highlight emerging strategies targeting the FGFR–RNA splicing bi-crosstalks, aiming to achieve precision theranostics. These include antisense oligonucleotides (ASOs) to correct aberrant splicing, soluble FGFR decoy receptors to block ligand engagement, and combinatorial regimens integrating FGFR tyrosine kinase inhibitors (TKIs) with spliceosome modulators to overcome resistance. Notably, isoform-specific PET/SPECT imaging probes enable patient stratification and real-time monitoring, providing a foundation for FGFR-driven radiotheranostics. In addition, novel modalities such as CRISPR/Cas9-based splicing modulation further expand the therapeutic landscape toward isoform-precise, mechanism-guided intervention, as well as rational combination therapies.

A deeper understanding of the FGFR-RNA splicing crosstalks will not only elucidate the molecular underpinnings of tumorigenesis but also accelerate the development of innovative therapeutic strategies, used alone or in combination, to overcome tumor plasticity and resistance.

## 2 FGFR-canonical signaling, physiological and pro-tumoral functions

### 2.1 FGFR structure and signaling initiation

FGFR consists of an extracellular ligand-binding domain, composed of three immunoglobulin-like domains (D1–D3), a single transmembrane helical region, and an intracellular tyrosine kinase (TK) domain with catalytic activity^14^. Upon ligand binding, FGFR undergoes a conformational change facilitated by heparan sulfate co-factors^15^, which alleviates autoinhibition mediated by the hinge region and activation loop. This structural rearrangement enables FGFR dimerization, a prerequisite for receptor activation^16^^,^^17^. Dimerized FGFR autophosphorylation consists in sequential phosphorylation of seven tyrosine residues inside the activation loop^18^. Among these, five key tyrosine residues are essential for activating and maintaining kinase function, with phosphorylation enhancing kinase activity by 50- to 1000-fold. In summary, FGFR structure and activation enable precise signal transduction, while their dysregulation drives cancer progression and therapeutic resistance.

### 2.2 Canonical FGFR signaling pathways and physiological functions

**Figure 1:**
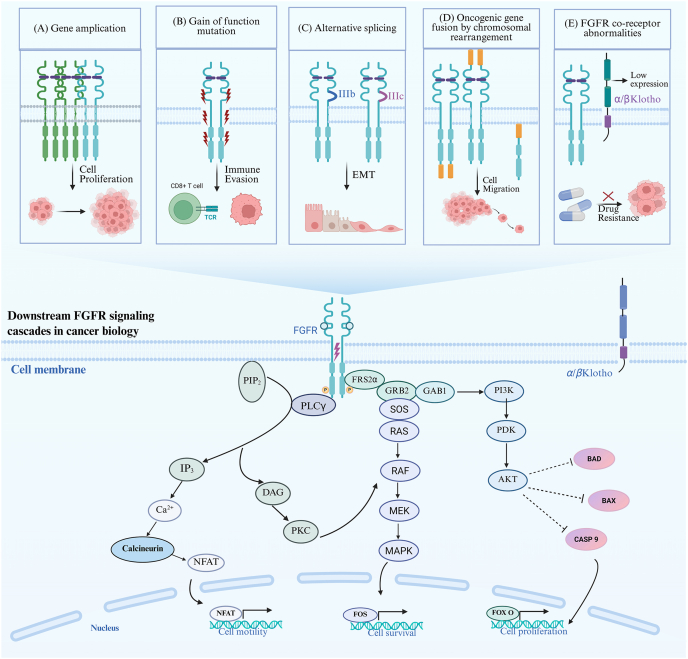
Mechanisms of FGFR dysregulation and downstream signaling pathways. (A) *FGFR* gene amplification promotes aberrant cell proliferation. (B) Gain-of-function mutations in *FGFR* facilitate immune evasion, as evidenced by disrupted interactions with CD8^+^ T cells *via* the T cell receptor (TCR). (C) Aberrant alternative splicing of FGFR drives EMT, enhancing cellular migratory capacity. (D) Oncogenic *FGFR* gene fusions, arising from chromosomal rearrangements, contribute to abnormal cell migration and tumor progression. (E) Dysfunction of FGFR co-receptors (*α*/*β*Klotho) results in decreased expression and is implicated in the development of drug resistance. The schematic pathway at the bottom illustrates key downstream signaling cascades activated by FGFR, including PLC*γ*, PI3K, MAPK. These pathways converge on molecular effectors such as BAD, BAX, and CASPASE-9, involving in cancer initiation, progression, and the development of drug resistance. Figure created with BioRender.

FGFRs phosphorylated residues serve as docking sites for scaffold adaptor proteins, which relay upstream signals to effectors and activate downstream signal transduction cascades (Fig. 1)^19^. Among them, FRS2 and phospholipase C*γ* (PLC*γ*) serve as key mediators that activate three canonical signaling cascades: the rat sarcoma/extracellular signal-regulated kinase–mitogen-activated protein kinase (RAS/ERK–MAPK) pathway, the phosphoinositide 3-kinase–protein kinase B (PI3K–AKT) pathway, and the PLC*γ* pathway^20^. FGFR-mediated activation of these pathways controls various critical biological processes, including cell proliferation, cell death, differentiation, angiogenesis and wound healing, which orchestrate cellular responses essential for tissue maintenance and regeneration^21^.

In terms of RAS/ERK–MAPK activation, FRS2 recruits the adaptor protein growth factor receptor-bound protein 2 (GRB2), which in turn associates with GRB2-associated binding protein 1 (GAB1) or the guanine nucleotide exchange factor Son of Sevenless (SOS), leading to the activation of RAS GTPase^22^. Activated RAS then initiates the MAPK signaling cascade, culminating in ERK activation. Additionally, FRS2 phosphorylation facilitates its interaction with the tyrosine phosphatase Src-homology protein tyrosine phosphatase 2 (SHP2), which enhances ERK activation, thereby promoting cellular processes such as proliferation, differentiation, and migration^23^^,^^24^.

The PI3K–AKT signaling axis is also activated downstream of FGFR through the adaptor complex comprising FRS2 and GRB2, which facilitates the recruitment of GAB1 (Fig. 1). Upon engagement, GAB1 interacts with and activates PI3K, initiating a signaling cascade that culminates in the phosphorylation and activation of AKT^25^. Activated AKT phosphorylates multiple downstream targets, including the pro-apoptotic B-cell lymphoma/leukemia-2 (Bcl-2) family member Bcl-2-associated death promoter (BAD), thereby inhibiting apoptosis and promoting cell survival ^26^. AKT, as well, modulates the activity of Forkhead box O (FOXO) transcription factors, which are key regulators of cell cycle progression, oxidative stress responses, and metabolic homeostasis^20^. In mesenchymal stem cells, FGFR-activation leads to the phosphorylation of downstream effectors ERK and AKT, which enhances cell proliferation, self-renewal, and pluripotency, as well as osteogenic and chondrogenic differentiation. Conversely, inhibition of ERK or AKT signaling significantly reduces cell proliferation and differentiation, underscoring the indispensable role of FGFR signaling in stem cell fate determination^27^.

In parallel, activated FGFR recruits and phosphorylates PLC*γ*1, which hydrolyzes PhosphatidylInositol-4,5-bisPhosphate (PIP2), generating Inositol-1,4,5-trisPhosphate (IP3) and DiAcylGlycerol (DAG) (Fig. 1)^28^. DAG activates protein kinase C (PKC), which phosphorylates substrates such as myristoylated alanine-rich C kinase substrate (MARCKS), influencing cell division, differentiation, and migration^29^. Meanwhile, IP3 promotes calcium ion release from intracellular stores, activating calcium-dependent proteins, including calcineurin^30^. Calcineurin, through the calcineurin/the nuclear factor of activated T cell (CaN/NFAT) signaling axis, regulates T cell activation and proliferation^31^. Another typical process highly regulated by FGFR is angiogenesis, a complex process involving endothelial cell activation, basement membrane degradation, endothelial proliferation and migration, and the formation of capillary networks. Activation of FGFR on endothelial cells triggers intracellular signaling pathways, particularly the MAPK and PKC pathways, which promote endothelial cell mitosis, migration, and neovascularization^32^^,^^33^.

### 2.3 Dysregulation of canonical FGFR signaling in cancer

As discussed above, FGFR-driven signaling pathways coordinate fundamental physiological cellular processes. In addition, numerous studies have now highlighted FGFR-signaling dysregulation in cancer progression and therapeutic resistance (Fig. 1). Dysregulation of FGFR, through mechanisms such as gene amplification, activating mutations, oncogenic fusions, or co-receptor (*α*/*β*Klotho) dysfunction, drives uncontrolled proliferation, immune evasion, epithelial–mesenchymal transition (EMT) and tumor migration, as well as therapeutic resistance. These alterations converge on canonical downstream cascades, including PLC*γ*, PI3K, and MAPK pathways, which further regulate apoptotic mediators (BAD, BAX, CASPASE-9), collectively sustaining cancer initiation, progression, and treatment resistance. Importantly, aberrant FGFR splicing can also contribute to dysregulation of FGFR signaling as elaborated in the following sections.

## 3 RNA alternative splicing as a driver of FGFR isoform diversity

Accumulating evidence suggests that the functional diversity and context-specific activity of FGFRs cannot be fully explained by genomic alterations or ligand variation alone. Hence, alternative splicing of *FGFR* pre-mRNA has now emerged as a critical regulator of FGFR signaling through the generation of various FGFR splice isoforms with structural and functional diversity, enabling FGFRs to regulate downstream signaling in a tissue- and disease-specific manner. A key functional consequence of this isoform diversity is the shift in ligand-binding selectivity, which in turn dictates downstream signaling outcomes (Table 1)^34^, ^35^, ^36^. This regulation is particularly relevant in cancer, where aberrant splicing of FGFRs contributes to EMT, therapy resistance, and metastatic progression. Thereby, deciphering the specificity and differential roles of FGFR splice isoforms is crucial for understanding their contribution to tissue maintenance and disease progression, as well as for developing precise therapeutic strategies notably in cancer. However, despite recent advancements in identifying the upstream factors and signaling pathways that influence FGFR splicing, the molecular mechanisms involved remain largely unexplored. The following sections (*i*.*e*., 3 and 4) aim to provide an “up to date” overview of how RNA splicing shapes FGFRs function and signaling specificity across both physiological and pathological contexts.

**Table 1:** Binding preferences of human FGF ligands for distinct FGFR splice isoforms, and cofactor/coreceptor requirements^34^, ^35^, ^36^.

FGF ligand	Subfamily	Cofactor/coreceptor requirement	FGFR isoform binding preference
FGF1 (aFGF)	Canonical	Heparan sulfate (HS)	All
FGF2 (bFGF)	Canonical	HS	FGFR1-IIIc = FGFR3-IIIc > FGFR2-IIIc = FGFR1-IIIb = FGFR4
FGF3	Canonical	HS	FGFR2-IIIb > FGFR1-IIIb
FGF4	Canonical	HS	FGFR1-IIIc = FGFR2-IIIc > FGFR3-IIIc = FGFR4
FGF5	Canonical	HS	FGFR1-IIIc = FGFR2-IIIc > FGFR3-IIIc = FGFR4
FGF6	Canonical	HS	FGFR1-IIIc = FGFR2-IIIc > FGFR3-IIIc = FGFR4
FGF7 (KGF)	Canonical	HS	FGFR2-IIIb > FGFR1-IIIb
FGF8	Canonical	HS	FGFR3-IIIc > FGFR4 >FGFR 2-IIIc = FGFR1-IIIc
FGF9	Canonical	HS	FGFR3-IIIc > FGFR2-IIIc > FGFR1-IIIc = FGFR3-IIIb >> FGFR4
FGF10	Canonical	HS	FGFR2-IIIb > FGFR1-IIIb
FGF11	Intracellular (non-secreted)	–	–
FGF12	Intracellular (non-secreted)	–	–
FGF13	Intracellular (non-secreted)	–	–
FGF14	Intracellular (non-secreted)	–	–
FGF15	Endocrine	*β*-Klotho	FGFR1-IIIc = FGFR4 > FGFR2-IIIc > FGFR3-IIIc
FGF16	Canonical	HS	FGFR3-IIIc = FGFR2-IIIc > FGFR1-IIIc = FGFR3-IIIb >> FGFR4
FGF17	Canonical	HS	FGFR3-IIIc = FGFR4 > FGFR2-IIIc = FGFR1-IIIc
FGF18	Canonical	HS	FGFR3-IIIc = FGFR4 > FGFR2-IIIc = FGFR1-IIIc
FGF19	Endocrine	*β*-Klotho	FGFR3-IIIc = FGFR4 > FGFR2-IIIc > FGFR1-IIIc
FGF20	Canonical	HS	FGFR3-IIIc = FGFR2-IIIc > FGFR1-IIIc = FGFR3-IIIb >> FGFR4
FGF21	Endocrine	*β*-Klotho	FGFR1-IIIc > FGFR3-IIIc > FGFR2-IIIc >> FGFR4
FGF22	Canonical	HS	FGFR2-IIIb > FGFR1-IIIb
FGF23	Endocrine	*β*-Klotho	FGFR1-IIIc = FGFR4 > FGFR3-IIIc > FGFR2-IIIc

### 3.1 Constitutive vs. alternative pre-mRNA splicing: definitions and general mechanisms

Pre-mRNA splicing is a fundamental process in eukaryotic gene expression, which contributes notably to regulating transcript diversity and proteome complexity. Constitutive splicing allows removal of introns and joining of exons to generate mature transcripts^37^. Alternative splicing is the process by which different transcripts, encoding sometimes distinct proteins, are generated from the same pre-mRNA. In humans, over 95% of multi-exon genes undergo alternative splicing in a developmental, tissue-specific, or signal transduction-dependent manner^38^. Such spatio-temporal and context-specific regulation allows alternative splicing to act as a key modulator of gene expression, playing crucial roles in biological processes such as cell differentiation and response to environmental changes^39^. Alternative splicing processing events include: skipped exon (SE), mutually exclusive exons (MXE), retained intron (RI), alternative 3′-splice site selection (A3SS), alternative 5′-splice site selection (A5SS), alternative first exon (AFE), alternative last exon (ALE) or tandem 3′ UTRs^40^. Dysregulation of alternative splicing is frequently observed in cancers and other diseases, highlighting its clinical relevance beyond basic gene regulation^41^^,^^42^.

**Figure 2:**
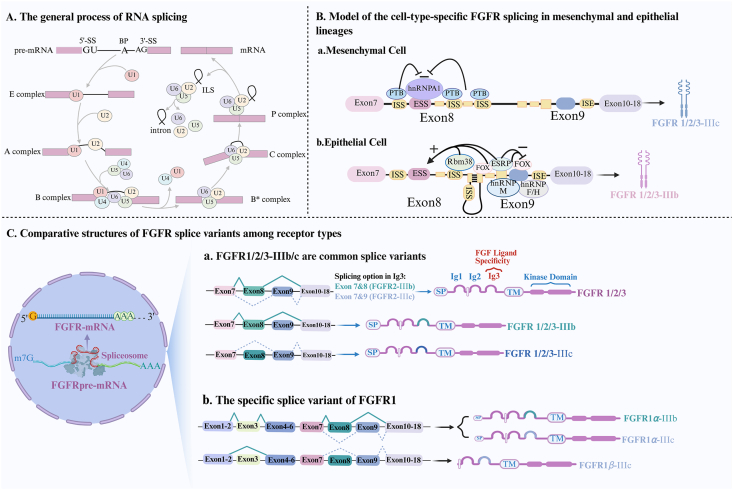
RNA splicing general mechanism and FGFR isoform diversity. (A) Schematic illustration of the general RNA splicing process. Precursor mRNA (pre-mRNA) is processed by the spliceosome to remove introns and ligate exons, resulting in mature mRNA transcripts. (B) Cell type-specific model of FGFR2 alternative splicing. In mesenchymal cells, the splicing machinery preferentially includes exon 9, producing the FGFR2-IIIc isoform, whereas in epithelial cells, exon 8 is incorporated, generating the FGFR2-IIIb isoform. (C) Structural comparison of FGFR splice variants (FGFR1-3), focusing on alternative splicing events within the immunoglobulin-like domain III (Ig-III), which generate IIIb and IIIc isoforms. Alternative inclusion/exclusion of *α*-exon (exon 3) creates two splicing forms, FGFR1*α* and FGFR1*β*, respectively. These isoforms differ in their ligand-binding specificity and exhibit distinct affinities for FGF ligands. Additionally, variations in the transmembrane and intracellular domains further contribute to differential receptor localization, signaling properties, and specific functions. Figure created with BioRender.

Constitutive and alternative pre-mRNA splicing is orchestrated by the spliceosome machinery that recognizes specific splice sites on pre-mRNA and allow excision of introns and joining of exons. The spliceosome is composed of five small nuclear RNAs (snRNAs)—U1, U2, U4, U5, and U6—along with approximately 100 associated proteins. U6 snRNA directly participates in catalysis within the nucleus, whereas other snRNAs are first processed in the cytoplasm before returning to the nucleus to facilitate splicing^43^. During the different steps of splicing reaction, distinct and highly dynamic small nuclear ribonucleoprotein (snRNP) complexes form in a timely and coordinated manner (Fig. 2A)^44^^,^^45^. These steps that include assembly, activation, catalysis, and disassembly, involve multiple spliceosome conformations, from early E complex and activated A spliceosomes to catalytic B, C complex and post-catalytic (P, ILS) states^46^. Initially, U1 snRNP binds the 5′ splice site (5′SS), while U2 snRNP, aided by U2AF, recognizes the branch point sequence (BPS) and the 3′ splice site (3′SS), forming the early spliceosome (E). The addition of the U4/U6/U5 tri-snRNP leads to a pre-catalytic conformation (B), which undergoes further rearrangement to activate splicing^47^^,^^48^. The catalytic phase involves two sequential transesterification reactions: the first reaction cleaves the 5′ exon and forms an intermediate lariat, while the second reaction ligates the exons, generating a mature mRNA and releasing the intron as a lariat^49^. Ultimately, spliceosome disassembly recycles its components for subsequent splicing cycles^50^, ^51^, ^52^.

Splicing factors further regulate splice site selection, ensuring both the fidelity and efficiency of pre-mRNA processing^53^. During alternative splicing, the binding of splicing factors, such as Ser/Rich-Arginine (SR) or heterogeneous nuclear ribonucleoproteins (hnRNP) proteins, to specific regulatory exonic (ESS, ESE) or intronic (ISS, ISE) elements contributes to the definition and recognition of exons and their inclusion/exclusion in the mature mRNA which depends on the relative expression and activity balance of these splicing factors.

### 3.2 FGFR isoform diversity generated by alternative splicing and its impact on downstream signaling

Alternative splicing of *FGFR* pre-mRNA has been shown to generate various receptor isoforms with distinct structural and functional properties^19^. A summary of the structural features and diversity of reported FGFR splice variants is presented in Fig. 2C. These splicing processing events impact various functional domains of FGFRs 1–3 mainly.

A well-characterized splicing event involves the mutually exclusive inclusion of exon 8 or 9 within the immunoglobulin-like domain III (IgIII) (Fig. 2B), producing the IIIb and IIIc isoforms, respectively^19^^,^^54^. Regulation of this splicing event is mediated by various RNA-binding proteins (RBPs) depending on the context. hnRNP M promotes exon IIIc skipping, favoring the production of FGFR2-IIIb^55^. Esophageal splicing regulatory protein 1 (ESRP1) and Esophageal splicing regulatory protein 2 (ESRP2) as well as hnRNP F/H and forkhead box-2 enhance the expression of the IIIb isoform. Conversely, hnRNP A1 and polypyrimidine tract-binding protein 1 (PTBP1) suppress the expression of IIIb isoform^55^. Additionally, RNA-binding motif protein 4 (RBM4) enhances IIIb expression, whereas neuronal PTB (nPTB) facilitates IIIc expression^56^. The precise balance of these splicing regulators is therefore essential for maintaining FGFR function and tissue-specific expression patterns of FGFRs. Beyond this classical model related to the expression of splicing factors, recent studies have demonstrated that dynamic histone modifications also play a critical role in modulating FGFR2 splicing towards the mesenchymal FGFR2 IIIc isoform^57^. Hence, it has been shown that AKT signaling regulates FGFR2 splicing by phosphorylating IWS1, facilitating the recruitment of histone methyltransferase SET-domain containing protein 2 and triggering H3K36me3 modifications. This modification recruits PTB, which inhibits exon IIIb inclusion while promoting exon IIIc inclusion^58^. In an EMT-induced cellular model, alterations in H3K27ac/me3 levels have also been shown to be both necessary and sufficient to drive FGFR2 IIIb/IIIc splicing shifts, as these chromatin modifications regulate PTB recruitment to pre-mRNA^59^. Localized histone modifications at chromatin-marked exons thus serve as a regulatory mechanism, driving the dynamic changes in FGFR2 alternative splicing.

Beyond the IgIII domain, alternative splicing events affect the extracellular region of FGFRs. For instance, exons 3 and 4 in FGFRs 1–3, and exons 2 and 3 in FGFR4, encode the IgI and acid box domains respectively, which contribute to FGFR auto-inhibition^54^^,^^60^. FGFR splicing isoforms lacking these domains exhibit enhanced ligand affinity and signaling potency^61^. Specifically, removal of the IgI domain generates the FGFR*β* isoform, which retains IgII and IgIII, forming two structural loops, whereas the FGFR*α* isoform, containing the IgI domain, maintains three loops (Fig. 2C)^62^. The regulation of these splicing events is mediated by PTBP1, which functions as a splicing repressor. PTBP1 expression levels directly influence FGFR1 splicing patterns. Overexpression of PTBP1 suppresses the inclusion of the *α* exon, whereas its downregulation enhances *α* exon retention. This regulation occurs *via* PTBP1 binding to intronic splicing silencers (ISS-1) flanking the *α* exon^63^^,^^64^. Additionally, serine/arginine-rich splicing factor 6 (SRSF6) facilitates the exclusion of the *α* exon by binding to exonic splicing enhancers (ESEs) within this region^64^. A summary of splicing regulators and RBPs involved in FGFR alternative splicing is presented in Table 2 ^56^^,^^63^^,^^65^, ^66^, ^67^, ^68^, ^69^, ^70^, ^71^, ^72^, ^73^, ^74^, ^75^, ^76^, ^77^, ^78^, ^79^, ^80^, ^81^, ^82^, ^83^, ^84^, ^85^, ^86^, ^87^, ^88^, ^89^, ^90^, ^91^, ^92^, ^93^, ^94^.

**Table 2:** Preferential expression and functional roles of various FGFR splice variants across tumor types, and key RNA-binding proteins (RBPs) and factors driving their splicing and expression regulation.

FGFR	Splice variants	Reported in tumors	RBPs and regulators that modulate RNA splicing and expression regulation	Phenotypes/functions associated with FGFR splice variants	Ref
FGFR1	FGFR1*α*, FGFR1*β*, FGFR1-IIIb, FGFR1-IIIc, FGFR1v	Breast cancer; Bladder cancer; Glioblastoma; High-grade diffuse glioma; Adenoid cystic carcinoma; Pancreatic ductal adenocarcinoma	ESRP1, ESRP2, PTBP1, SRSF6 (SRp55)	• Proliferation: FGFR1*β* enhanced growth in bladder cancer; • Migration/metastasis: High FGFR1*β* is associated with increased spread in breast cancer, bladder cancer and high-grade diffuse glioma; FGFR1-IIIc drives EMT-associated invasion and metastasis; • Drug sensitivity: High FGFR1*β* improves response to FGFR-TKIs inhibitors; • Drug resistance: FGFR1v promotes AXL/AKT-mediated resistance; • Tumor suppression: FGFR1-IIIb reduces proliferation, migration, invasion, and tumorigenicity	63,65, 66, 67, 68, 69, 70, 71, 72, 73
FGFR2	FGFR2-IIIb, FGFR2-IIIc	Cholangiocarcinoma; Gastric cancer, Bladder Cancer; Endometrial cancer; Colorectal cancer; Pancreatic cancer; Esophageal squamous cell carcinoma; Diffuse gastric cancer; Breast cancer	ESRP1/2, hnRNP F, RBM4, nPTB	• Proliferation: FGFR2-IIIb enhanced growth in cancers with its ligands likely FGF7/FGF10 overexpression • EMT & metastasis: FGFR2-IIIc drives EMT and metastasis; • Invasiveness: FGFR2-IIIc enhances tumor aggressiveness; • Drug resistance: FGFR2-IIIc promotes resistance to anti-FGFR2-IIIb antibodies; • Prognosis: FGFR2-IIIc causes poor survival in diffuse gastric cancer	56,74, 75, 76, 77, 78, 79
FGFR3	FGFR3-IIIb, FGFR3-IIIc, FGFR3-S, FGFR3-L, FGFR3 DAB, FGFR3Δ8–10	Colorectal cancer; Prostate cancer; Liver cancer; Esophageal cancer; Bladder cancer	hnRNP F, LncRNA-UCA1	• Proliferation: FGFR3-S drives prostate tumor growth; • Invasiveness & resistance: FGFR 3-IIIc enhances tumor aggressiveness; FGFR3-S enhances resistance to dovitinib in prostate cancer; • Sustained signaling: FGFR3Δ8–10 loss leads to sustained FGFR3 signaling in bladder cancer; • Mitogenic responses: FGFR3 DAB enhances downstream signaling;	80, 81, 82, 83, 84, 85, 86, 87, 88, 89, 90
FGFR4	(Not well-characterized)	Pancreatic cancer; Colorectal cancer; Hepatocellular carcinoma	FOXC1, SNRPE, HOXB5	• Proliferation: Drives cancer cell growth; • Metastasis: Promotes invasion in colorectal cancer; • Oncogenic loop: FGF19–HOXB5–FGFR4 axis fuels hepatocellular carcinoma progression; • RNA surveillance activation: SNRPE knockdown triggers nonsense-mediated decay, reduces FGFR4 levels, and suppresses tumorigenesis and progression	91, 92, 93, 94

Additional FGFRs splice variants include alternative C-terminal sequences (C1, C2, and C3) which modulate receptor activity. These C-terminal variations impact key tyrosine residues involved in receptor autophosphorylation and interaction with cytoplasmic proteins that trigger FGFR downstream signaling. Among these, the C3 isoform exhibits the strongest transforming activity and oncogenic potential. Elevated C3 isoform expression has been reported in gastric and breast cancer cell lines, reinforcing its role in tumorigenesis^95^.

Another splicing event affecting FGFR function involves the inclusion or exclusion of a six-nucleotide sequence (GTAACA) near the membrane-spanning domain, referred to as the VT motif^96^. Inclusion of the VT motif enhances RAS/MAPK pathway activation, whereas its exclusion impairs effector protein binding, thereby attenuating receptor signaling. Interestingly, while VT motif exclusion disrupts major FGFR downstream pathways, certain kinase-dependent signaling cascades, such as those mediated by PLC*γ*, remain unaffected. This suggests that VT motif inclusion may confer cell type-specific signaling preferences^96^.

### 3.3 Isoform-specific alternative splicing of FGFRs alters FGF ligand binding preference

Alternative splicing of FGFRs not only dictates their tissue- and cell type-specific expression but also determines their ligand-binding preferences. For instance, FGFR2-IIIb preferentially binds FGF1, 3, 7, 10, and 22, whereas FGFR2-IIIc has higher affinity for FGF1, 2, 4, 5, 6, 8, 9, 16, 17, 18, and 20^36^^,^^97^^,^^98^ (Table 1). Similarly, FGFR1*β* shows enhanced binding to FGF1^54^. Such isoform-specific binding differences have critical functional consequences.

**Tissue repair:** In response to skin injury, fibroblasts within granulation tissue and *γδ*T cells in the dermis and epidermis upregulate the expression of FGF7 and FGF22, which activate FGFR2-IIIb in keratinocytes, promoting their proliferation and migration. Additionally, fibroblasts secrete FGF7 and FGF10, which signal through FGFR1-IIIb and FGFR2-IIIb to drive basal keratinocyte proliferation and migration, thereby facilitating re-epithelialization. Keratinocytes further enhance this process by secreting FGF22 in an autocrine manner, sustaining their activation and accelerating wound closure^99^.

**Organ development:** During lung development, epithelial–mesenchymal interactions mediated by FGF signaling are essential^100^. FGF10, a key regulator of early lung branching morphogenesis, is required for proper lung formation. In FGF10-knockout mice, tracheal development occurs, but lung bud formation is absent. Mesenchymal FGF10 signaling through epithelial FGFR2 activates the MAPK/ERK pathway, promoting SRY-box 9 (SOX9) expression and lung epithelial differentiation^101^. FGFR2-IIIb, which has a high affinity for FGF10, further regulates AKT and WNT/*β*-catenin signaling, influencing lung progenitor cell fate decisions. Although airway and alveolar epithelial cells differ functionally, they originate from common embryonic epithelial progenitors, which form the respiratory tree and differentiate into mature lung lineages^102^^,^^103^. FGF10–FGFR2-IIIb signaling plays a crucial role in proximal-to-distal airway patterning by modulating the AKT–WNT/*β*-catenin axis. Loss of FGF10 or FGFR2-IIIb disrupts distal progenitor cell maintenance, leading to aberrant proximal lung differentiation, impaired surfactant production, and alveolar epithelial cell lineage defects, potentially contributing to respiratory disorders.

Together, above examples illustrate how isoform-specific FGFR–FGF interactions govern context-dependent outcomes, ranging from skin repair to lung development. Disruptions in these splicing-ligand relationships can lead to pathological conditions such as defective tissue regeneration and respiratory disorders, underscoring the functional diversity of FGFs and their splice isoform-specific receptors across different biological settings.

### 3.4 External cues driving FGFR splicing

Increasing studies highlight that external factors such as viral infections^104^, hormonal stimulation^105^, cellular stresses^105^, and circadian rhythm regulation^106^ could direct influence the alternative splicing of FGFR, particularly FGFR2.

For instance, in esophageal squamous cell carcinoma (ESCC), exposure to the carcinogen *N*-nitroso-methylbenzylamine (NMBA) results in the downregulation of the long non-coding RNA UCA1, which functions to sequester the splicing factor hnRNP F. Loss of UCA1 allows hnRNP F to bind *FGFR2* pre-mRNA at intronic regions flanking exon IIIc, thereby promoting its inclusion and facilitating a switch from the epithelial isoform (IIIb) to the mesenchymal isoform (IIIc). This isoform switch activates the PI3K/AKT signaling cascade and drives EMT^107^. These findings directly link environmental mutagens to RBPs-mediated alternative splicing of FGFR2 in carcinogenesis.

Moreover, cellular stress signals such as FAS activation have been shown to regulate FGFR2 splicing through a protein–protein interaction network involving the FAS-activated serine/threonine phosphoprotein (FAST). FAST itself lacks RNA-binding capacity but acts as a scaffold that recruits splicing regulators, including T-cell intracellular antigen-1 (TIA1), SAM68, and SF3B4, to U-rich intronic splicing enhancers (ISEs) located adjacent to exon IIIb of FGFR2. This assembly facilitates exon definition and enhances IIIb inclusion, counterbalancing EMT-promoting isoforms and modulating tumor cell plasticity under stress conditions^108^.

In addition, alterations in circadian rhythm components can profoundly affect the temporal regulation of splicing factors and downstream targets such as FGFR2. Knockdown of core circadian genes, *ARNTL*, *PER2*, and *NR1D1*, in HCT116 colon cancer cells disrupts the rhythmic expression of critical spliceosome components (*e*.*g*., SF3A1, SNW1, HNRNPC) and the U2 auxiliary factor U2AF1. This deregulation is accompanied by a loss of periodicity in FGFR2 IIIb and IIIc isoform expression^106^. Mechanistically, ARNTL depletion reduces promoter activity and chromatin accessibility at loci encoding clock-controlled RBPs, thereby attenuating their ability to regulate exon selection. The resultant shift in the FGFR2 IIIb/IIIc ratio contributes to EMT signaling, enhancing tumor cell invasion and metastasis. These findings firstly highlight the circadian regulation of FGFR splicing as a potential contributor to cancer progression. Nevertheless, more detailed molecular studies are required to elucidate how circadian clock factors such as ARNTL coordinate with RBPs to regulate *FGFR* pre-mRNA splicing, as the current evidence is largely based on functional knockdown assays.

Although these studies focused on FGFR2 IIIb/IIIc splicing switch, a broad range of external stimuli, including inflammatory cytokines and growth factors, hypoxia and oxidative stress, extracellular matrix (ECM) remodeling and mechanical stress, chemotherapy/radiotherapy, and targeted therapies, may similarly modulate alternative splicing of additional FGFRs. Further research is warranted to explore whether these regulatory pathways are universally applicable across different tumor contexts and therapeutic settings.

## 4 Splicing-driven FGFR isoform diversity in tumorigenesis

**Figure 3:**
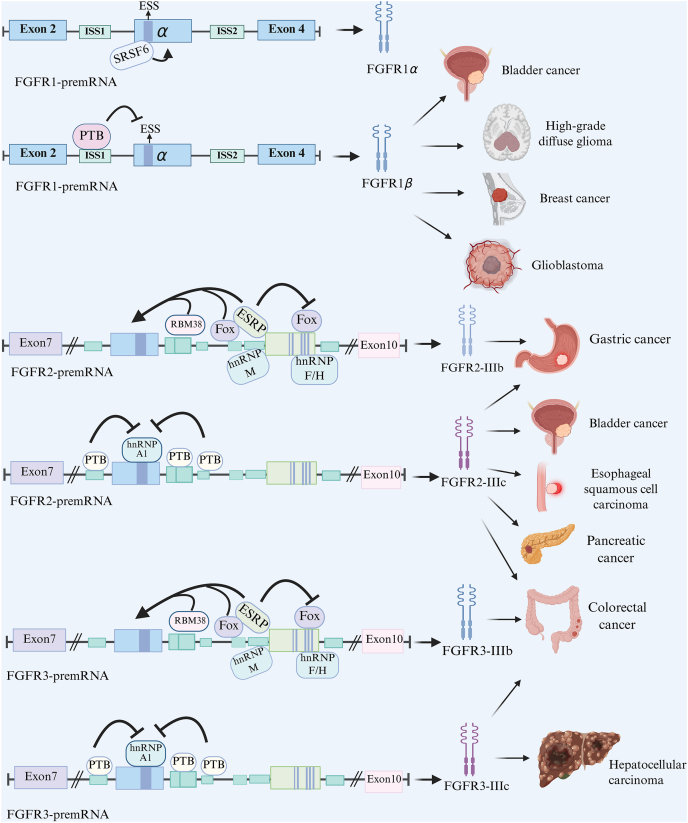
Different FGFR splicing isoforms exhibit cancer type-specific expression patterns; For example, FGFR1*β* is preferentially expressed over FGFR1*α* in breast cancer, bladder cancer, glioblastoma, and high-grade diffuse glioma. Additional isoform distributions are detailed in the main text. The figure also illustrates RNA-binding proteins and splicing factors that regulate these isoform-specific splicing events. Figure created with BioRender.

Extensive studies have demonstrated that dysregulated FGFR splicing also occurs across multiple cancer types (Fig. 3), altering receptor isoform expression and reprogramming downstream oncogenic pathways^109^. In this section, we systematically classify distinct FGFR splice isoforms and delineate their roles in driving specific type of cancer progression, metastasis, and therapeutic resistance (Table 2 ^56^^,^^63^^,^^65^, ^66^, ^67^, ^68^, ^69^, ^70^, ^71^, ^72^, ^73^, ^74^, ^75^, ^76^, ^77^, ^78^, ^79^, ^80^, ^81^, ^82^, ^83^, ^84^, ^85^, ^86^, ^87^, ^88^, ^89^, ^90^, ^91^, ^92^, ^93^, ^94^). This overview highlights the central contribution of the FGFR-splicing axis to tumor plasticity and adaptive progression.

### 4.1 FGFR1 splice variants across solid tumors: functions and therapeutic implications

FGFR1*β* displays stronger binding affinity for FGF1 than FGFR1*α*, thereby amplifying oncogenic signaling, promoting EMT and metastasis, and correlating with poorer patient survival. Paradoxically, high FGFR1*β* expression also increases tumor sensitivity to FGFR inhibitors, underscoring its dual role in driving tumor progression while enhancing therapeutic responsiveness^65^.

Breast cancer remains a major global health burden, accounting for approximately 30% of all female malignancies and contributing significantly to cancer-related mortality in women^110^. In these tumors, Zhao et al.^63^ demonstrated that FGFR1*β* activation enhances PI3K/S6 signaling, promoting proliferation, and represses E-cadherin expression, facilitating EMT. A high FGFR1*β*/FGFR1*α* ratio has been associated with increased invasiveness and altered transcriptional programs driving breast cancer progression.

In bladder cancer, FGFR1*β* expression is significantly upregulated in tumor tissues and cell lines, where it correlates with advanced pathological grade, stage, and poor clinical outcome. FGFR1*β* promotes proliferation, migration, and invasion *via* MAPK pathway activation, contributing to a more aggressive tumor phenotype^63^^,^^65^. FGFR1*β* is thus emerging as a prognostic biomarker and potential therapeutic target. Approaches such as selective FGFR1*β* inhibitors and isoform-specific antibodies are under investigation for personalized treatment strategies in bladder cancer.

In high-grade diffuse gliomas (HGG), aberrant FGFR1 splicing cooperates with NF1 splicing alterations to enhance tumor aggressiveness. Inclusion of NF1 exon 23a augments RAS/MAPK signaling independently of canonical mutations, while elevated FGFR1*β* expression further amplifies FGF-driven signaling^66^. These combined splicing events promote dedifferentiation, proliferation, and invasiveness. Therapeutically, simultaneous targeting of FGFR1*β* and NF1-associated pathways, for example through c-Jun N-terminal kinase (JNK) inhibition, may help restore RAS pathway regulation and improve treatment outcomes.

In pancreatic ductal adenocarcinoma, enforced expression of FGFR1-IIIb exerts tumor-suppressive effects by inhibiting proliferation, migration, invasion, and xenograft growth, primarily through suppression of ERK1/2 activity alongside activation of p38 and JNK signaling^72^. By contrast, FGFR1-IIIc expression in the same context enhances proliferative and invasive potential, highlighting its pro-tumorigenic function. Mechanistically, studies in EMT-driven models have shown that TGF*β*–SMAD3 signaling induces the transcription factor Forkhead box protein 1 (FOXO1), which directly upregulates FGFR1-IIIc and drives invasion, whereas FOXO1 silencing markedly diminishes this effect^73^.

In adenoid cystic carcinoma (ACC), novel truncated FGFR1 splice variants (FGFR1v) lacking intracellular kinase domains have been detected^67^^,^^70^. Unlike canonical FGFR1, FGFR1v functions independently of FGF ligation and instead signals through the AXL/AKT axis, driving resistance to FGFR inhibitors such as Dovitinib and Bemcentinib (BGB324). Notably, dual blockade of FGFR1v and AXL demonstrates synergistic efficacy in preclinical models, revealing an alternative resistance mechanism and providing a strong rationale for combination therapies in ACC. Collectively, these findings underscore the functional heterogeneity and tumor-specific roles of FGFR1 splice variants.

### 4.2 FGFR2 isoform switching in cancer progression, EMT, and metastasis

Dysregulated splicing of FGFR2 contributes to tumor progression in multiple cancers, including gastric carcinoma and cholangiocarcinoma, where differential isoform expression influences both tumor growth and metastatic behavior^111^^,^^112^. Moreover, *FGFR2* gene amplification and aberrant splicing are frequently observed in gastric and bladder cancers and are associated with unfavorable prognosis^75^.

Interestingly, FGFR2-positive circulating tumor cells have recently emerged as a promising non-invasive biomarker in gastric cancer^74^. However, current studies do not distinguish FGFR2 isoforms within circulating tumor cells, which may limit their predictive and clinical utility. Evidence from tumor tissue studies underscores the importance of isoform-specific regulation in diffuse gastric cancer, where ESRP1 is frequently upregulated through gene amplification and promoter hypomethylation, thereby biasing splicing toward the FGFR2-IIIb isoform while suppressing FGFR2-IIIc expression^113^^,^^114^. Teles et al.^115^ provided the first clinical evidence that ESRP1 overexpression driven by gene amplification promotes FGFR2 isoform expression bias and demonstrated a direct association between elevated FGFR2-IIIc expression and poor prognosis in diffuse gastric cancer. These findings provide new insights into the failure of current FGFR2-targeted therapies. Notably, most existing FGFR2-targeted monoclonal antibodies, such as GP369, selectively recognize FGFR2-IIIb, highlighting the urgent need to develop FGFR2-IIIc-specific diagnostic and therapeutic strategies.

In pancreatic cancer, miR-23a is upregulated during EMT and suppresses ESRP1, leading to splicing shifts in both Cluster of Differentiation 44 (CD44) (from CD44v to CD44s) and FGFR2 (from IIIb to IIIc), thereby promoting metastasis^116^, ^117^, ^118^. Restoration of ESRP1 can partially reverse these effects, highlighting its central role in splicing regulation and EMT suppression. Given that FGFR2-IIIc is associated with aggressive tumor behavior and is not effectively targeted by current FGFR2-IIIb-selective therapies, a combinatorial approach targeting both FGFR2 splicing isoforms and the upstream miR-23a-ESRP1 axis may provide synergistic benefits, notably overcome isoform-driven resistance.

In colorectal cancer (CRC), the RNA-binding protein RBM4 modulates FGFR2 splicing in opposition to nPTB, forming a regulatory circuit that impacts EMT and angiogenesis^56^. Downregulation of RBM4, driven by miR-92a, leads to increased nPTB expression and a shift toward FGFR2-IIIc, thereby promoting tumor progression *via* AKT/ERK activation. Therapeutic restoration of RBM4 or inhibition of the miR-92a/nPTB axis may reverse these oncogenic effects.

FGFR2-IIIc also plays a prognostic role in endometrial cancer. Its overexpression correlates with poor progression-free and disease-specific survival, particularly in high-risk and MisMatch Repair-deficient endometrioid endometrial carcinoma (EEC) cases^78^. FGFR2-IIIc may serve as an independent prognostic biomarker in EEC.

Within the tumor microenvironment, tumor-associated macrophages (TAMs) can influence tumor dynamics through changing FGFR2 different isoform's expression. FGFR2-IIIc is regulated also by ESRP1/2 and contributes to TAM polarization^119^^,^^120^. Modulating the ESRP1/2–FGFR2-IIIc axis can affect TAM phenotype, potentially enhancing anti-tumor effects, particularly in breast cancer^76^.

In esophageal squamous cell carcinoma, carcinogen-induced downregulation of the long noncoding RNA UCA1 promotes a switch from FGFR2-IIIb to IIIc, thereby activating PI3K–AKT signaling and facilitating EMT^121^^,^^122^. Re-expression of UCA1 inhibits this transition and could offer a therapeutic avenue.

Collectively, these findings underscore the pathological significance of FGFR2 isoforms dynamics, particularly the mesenchymal-promoting FGFR2-IIIc. Isoform-specific inhibition, with upstream regulatory intervention (*e*.*g*., ESRP1, RBM4, or miR-23a), and RNA splicing inhibitors, represents a rational combinatorial strategy to suppress EMT and metastatic progression as well overcome splicing-driven resistance. Such strategies offer promising new opportunities for precision FGFR-targeted therapies.

### 4.3 FGFR3 splicing dysregulation in colorectal, prostate, and bladder cancers

Similar to *FGFR1* and *FGFR2 genes*, the *FGFR3* gene undergoes alternative splicing within the IgIII domain, generating two major isoforms: FGFR3-IIIb and FGFR3-IIIc^80^. These isoforms differ in ligand specificity and biological functions, thereby influencing tumor behavior. In CRC, aberrant FGFR3-IIIb expression has been linked to enhanced cell survival and proliferation, whereas FGFR3-IIIc acts as a principal mediator of FGF18-driven oncogenic signaling, promoting tumor growth and progression^81^^,^^85^. Importantly, selective inhibition of FGFR3-IIIc suppresses CRC cell proliferation and induces apoptosis, underscoring its potential as a therapeutic target. In hepatocellular carcinoma, FGF9 activation of both FGFR3-IIIb and IIIc isoforms stimulates tumor growth and neovascularization^86^. Together, these isoforms not only inhibit apoptosis but also sustain clonogenic growth, further accelerating malignant progression.

In prostate cancer, a novel splice variant, FGFR3-S, which lacks exon 14 encoding the activation loop of the kinase domain, exhibits increased autophosphorylation and constitutive activation of downstream pathways such as AKT, Signal transducer and activator of transcription 3 (STAT3), and S6 ribosomal protein. FGFR3-S enhances tumor growth and confers resistance to FGFR inhibitors such as dovitinib in xenograft models, compared to the full-length FGFR3-L isoform^82^.

Another variant, FGFR3-DAB, lacking the acidic box domain, displays enhanced responsiveness to FGF2 stimulation. The acidic box appears to modulate ligand binding and receptor activation, and its absence may contribute to increased mitogenic activity^87^. In the context of bladder cancer, *FGFR3* mutations and splicing alterations not only drive tumor progression but also shape an immunosuppressive tumor microenvironment. FGFR3 activation has been shown to promote macrophage-mediated immune evasion by suppressing T cell activation^88^. These findings underscore the dual role of FGFR3 splice variants in promoting both intrinsic tumor cell aggressiveness and extrinsic immune escape^89^. They highlight the therapeutic potential of targeting FGFR3 isoform-specific signaling to simultaneously restrain tumor growth and reprogram the tumor immune microenvironment.

In bladder tumors, expression of FGFR3Δ8–10, a splice variant that normally acts as a negative regulator of FGFR3 signaling, is markedly reduced^80^. Loss of this variant leads to constitutive FGFR3 pathway activation, thereby promoting tumor cell proliferation and resistance to apoptosis. At the same time, upregulation of the FGFR3-IIIc isoform broadens ligand-binding specificity and triggers multiple oncogenic pathways, further enhancing tumor aggressiveness. Clinically, therapeutic agents such as the selective FGFR inhibitor derazantinib and pan-FGFR inhibitor have shown promising efficacy in bladder cancers harboring FGFR3 alterations^90^. Collectively, these findings highlight the central role of FGFR3 splicing dysregulation in driving tumorigenesis and therapeutic vulnerability across colorectal, prostate, and bladder cancers.

### 4.4 FGFR4 in cancer metastasis: splicing-dependent and independent regulation distinct from FGFR1-3

Distinct from FGFR1–3, FGFR4 does not undergo alternative splicing to generate IIIb/IIIc isoforms, owing to the absence of alternative exons in its IgIII domain^123^. Its expression is finely regulated through multiple transcriptional and post-transcriptional mechanisms that contribute to tumor progression and metastasis.

In pancreatic cancer, Hepatocyte Nuclear Factor 1-alpha (HNF1*α*) has been shown to enhance intron 1 enhancer activity, leading to FGFR4 overexpression and promoting tumor aggressiveness^91^^,^^94^. Similarly, in metastatic CRC, the transcription factor FOXC1 transcriptionally activates FGFR4 and its downstream effector genes such as *ITGA7*, thereby facilitating EMT and metastatic dissemination *via* the GSK3*β*/*β*-catenin signaling axis^92^. Elevated FOXC1 expression correlates with increased CRC metastasis, recurrence, and reduced patient survival.

Beyond transcriptional control, emerging evidence highlights the contribution of post-transcriptional mechanisms in modulating FGFR4 expression. In hepatocellular carcinoma, small nuclear ribonucleoprotein polypeptide E (SNRPE) plays a critical role in regulating intron retention (IR) within FGFR4 transcripts, thereby influencing their stability through nonsense-mediated decay (NMD)^93^. Depletion of SNRPE increases intron retention, resulting in non-functional FGFR4 transcripts that are degraded *via* NMD, ultimately reducing FGFR4 protein levels. Functionally, suppression of FGFR4 through this splicing-dependent mechanism impairs HCC cell proliferation, migration, and clonogenicity, suggesting that modulation of the SNRPE–FGFR4 axis may offer therapeutic benefit.

Furthermore, a positive feedback loop involving FGF19, HOXB5, and FGFR4 has been identified in hepatocellular carcinoma, further amplifying oncogenic signaling. FGF19 stimulates HOXB5 expression *via* the PI3K/AKT/HIF-1*α* pathway, and HOXB5 in turn directly binds to the *FGFR4* promoter, sustaining high FGFR4 expression^91^. This FGF19–HOXB5–FGFR4 circuit drives hepatocellular carcinoma proliferation and metastasis.

Together, these findings delineate the multifaceted regulation of FGFR4 expression in pancreatic, colorectal, and liver cancers. They emphasize that although FGFR4 lacks canonical splicing isoforms, its expression and activity are tightly modulated by transcriptional enhancers, splicing factors, and oncogenic feedback circuits. Targeting these regulatory axes holds promise for controlling FGFR4-driven tumor metastasis, particularly in gastrointestinal and hepatocellular carcinoma malignancies.

## 5 FGFR signaling initiates downstream RNA splicing programs involved in tumorigenesis

While sections 3 and 4 focused on how RNA splicing contributes to FGFRs splice isoforms diversity and impacts by the way FGFRs functions, FGFRs themselves can also shape splicing outcomes through at least two complementary mechanisms. First, specific *FGFR* mutations can disrupt *cis*-acting splice elements, alter exon inclusion or exclusion and thereby driving disease phenotypes. Second, even though it remains largely unexplored, it is highly plausible that aberrant FGF-FGFR signaling cascades reprogram splicing regulatory networks globally.

Beyond altering receptor activity, *FGFR* mutations can profoundly affect pre-mRNA splicing regulation, thereby reshaping receptor isoform expression and downstream signaling^124^. For example, FGFR2 splicing is tightly regulated by *cis*-acting elements, including intronic splicing silencers (ISSs)^125^. Mutations in these regulatory elements can result in aberrant exon inclusion or exclusion, thereby leading to altered receptor signaling specificity and strength. Hence, mutations in *FGFR2* frequently disrupt the mutually exclusive selection of exons IIIb and IIIc, leading to misregulated epithelial–mesenchymal interaction and defective morphogenesis^126^^,^^127^. Deleting the IIIc exon, together with intronic sequences, caused splicing defects and an Apert syndrome-like dominant-lethal phenotype^126^. Similarly, the FGFR2p.C342Y mutation has also been shown to cause splicing abnormalities^128^. This mutation promotes abnormal exon usage and enhances the expression of the FGFR2-IIIc isoform, leading to significant craniofacial malformations, including craniosynostosis, midface hypoplasia, and behavioral deficits, without affecting limb development. Mutations can also impact other FGFRs, synonymous mutations in *FGFR1* can activate hidden splice sites in exon 8 and cause abnormal splicing, which may lead to congenital diseases such as Hartsfield and Kallmann syndromes. These pathologies are often associated with symptoms like loss of smell, problems in reproductive organ development, and abnormalities in the central nervous system^129^, ^130^, ^131^.

In addition to these mutation-driven alterations in *cis*-regulatory splicing elements, there is growing interest in how FGFR-mediated signaling pathways may influence splicing outcomes at the post-transcriptional level. Rather than altering splice site architecture directly, FGFR activation can modulate the activity of splicing regulators through downstream effectors, such as protein kinases. This signaling-based control introduces an additional layer of complexity that integrates extracellular cues together with the splicing machinery, thereby fine-tuning isoform expression in response to environmental and oncogenic stimuli. The following section explores current evidence supporting this indirect regulatory axis and its functional relevance in cancer biology.

To the best of our knowledge, direct experimental evidence linking FGFR signaling to RNA splicing remains limited. However, it is mechanistically plausible, particularly *via* the AKT pathway, that FGFR signaling modulates splicing regulation. This notion is supported by the well-characterized epidermal growth factor receptor (EGFR)–AKT–SRPK/SRSF axis, where Serine/arginine-Rich Protein Kinases (SRPKs), especially SRPK1, phosphorylate Serine/arginine-Rich splicing factors (SRSFs), thereby regulating their nuclear localization and splicing activity^132^, ^133^, ^134^. The EGFR–AKT–SRPK–SRSF signaling cascade links extracellular growth factor signals to splicing regulation, where EGFR activation leads to AKT-mediated activation of SRPK1, which then phosphorylates serine/arginine-rich proteins (SR proteins) leading to widespread changes in splicing^135^.

Given the shared downstream effectors between EGFR and FGFR pathways, it is reasonable to propose that FGFR-driven activation of AKT similarly engages the SRPK–SRSF cascades. This axis may orchestrate oncogenic splicing programs by modifying SR proteins and other RBPs, such as hnRNPs. Supporting this, phosphorylation of hnRNP L has been shown to increase its affinity for exon 3 of caspase-9b (C9/E3), promoting a shift toward the anti-apoptotic caspase-9b isoform and reducing the pro-apoptotic caspase-9a variant, thus enhancing cancer cell survival^136^.

Additionally, AKT activates SRPKs, which in turn mediate the nuclear translocation of splicing factors by facilitating their transition from heat shock protein 70 (HSP70)-bound complexes to heat shock protein 90 (HSP90)-bound complexes. This shift enhances SR proteins phosphorylation and ultimately modulates RNA splicing patterns. Notably, many splicing factors regulated by the AKT pathway exhibit either oncogenic or tumor-suppressive functions, highlighting the potential of targeting splicing factor pathways as a therapeutic strategy in cancer^135^. Heightened AKT phosphorylation correlates with increased SRPK1 levels, which enhances cancer cell resistance to apoptosis^137^.

Splicing regulators and kinases can be modulated by various signaling molecules, including growth factors or cytokines such as epidermal growth factor (EGF), platelet-derived growth factor, TGF*β*^138^, ^139^, ^140^, ^141^. Our previous work demonstrated that FGF-2 promotes angiogenesis by activating an SRSF1/SRSF3/SRPK1 network that regulates vascular endothelial growth factor receptor 1 *(VEGFR1)* mRNA alternative splicing in endothelial cells, a process that may also contribute to lung tumor progression. Upon FGF-2 activation of FGFR, SRSF1 and SRPK1 expression are upregulated, leading to the production of specific VEGFR1 splice variants, with sVEGFR1-ex12 being the most potent in enhancing angiogenesis^142^. Inhibition of FGFR with tyrosine kinase inhibitors or blocking SRPK1/2 kinase activities using SPHINX31 or SRPIN340 effectively blocks the FGF2-induced production of the pro-angiogenic splice variant VEGFR1-ex12, thereby indirectly suppressing angiogenesis. Notably, sVEGFR1-ex12 serves as a biomarker of poor prognosis in squamous lung carcinoma patients.

The RNA-binding protein Src-associated in mitosis 68 (SAM68), a member of the STAR family, plays a central role in RNA transcription, processing, and EGF signaling. EGF activates the ERK-MAPK pathway, leading to phosphorylation of SAM68, which regulates the alternative splicing of CD44, a key mediator of tumor progression^143^. Given the similarity between EGFR and FGFR signaling pathways, both of which involve receptor tyrosine kinase activation and PI3K–AKT and ERK–MAPK cascades^25^^,^^144^, it is plausible that FGFR signaling mobilizes a similar complex regulatory network to control RNA splicing regulatory programs contributing to tumor initiation and progression. As noted by Prof. Xiang-Dong Fu, "*it appears that EGF mobilizes a large village to accomplish regulated splicing during tumor progression*"^145^. Whether FGF is also part of this village remains to be deepened. This is crucial as unraveling intricate splicing mechanisms linked to FGF–FGFR-driven oncogenesis could help to define potential therapeutic targets for diagnosis improvements and definition of novel cancer treatment, as discussed in the following section.

## 6 Targeting FGFR-splicing crosstalks: biomarker discovery and therapeutic innovation

### 6.1 Clinical challenges of current therapies based on FGFR-TKIs

FGFR-TKIs can be broadly categorized based on their target selectivity: pan-FGFR inhibitors (*e*.*g*., erdafitinib, futibatinib, rogaratinib), FGFR1-3 selective inhibitors (*e*.*g*., pemigatinib), and isoform-specific agents (*e*.*g*., LOXO-435 for FGFR3, Fisogatinib for FGFR4)^10^ (Table 3). FGFR-TKIs have received regulatory approval and have shown efficacy in various solid tumors. For example, erdafitinib, the first FDA-approved pan-FGFR inhibitor, demonstrated an objective response rate (ORR) of 40% and a median progression-free survival (PFS) of 5.5 months in metastatic urothelial carcinoma^146^. Pemigatinib achieved an ORR of 35.5% and extended median overall survival (OS) to 21.7 months in cholangiocarcinoma patients with FGFR2 fusions^147^. Additionally, KIN-3248 has exhibited anti-tumor activity in FGFR2/3-driven advanced solid tumors, though its optimal clinical dose remains undetermined^148^. Furthermore, recent evidence suggested that FGFR4 inhibitor lenvatinib enhances antitumor immune responses in gastric cancer by suppressing the FGF19–FGFR4 signaling axis^149^, thereby reducing tumor programmed cell death-ligand1 expression and modifying the tumor immune microenvironment. This provides a new approach for the combined use of FGFR inhibitors and immune checkpoint inhibitors to combat drug resistance.

**Table 3:** Different types and status of FGFR-TKIs in indicated cancer types.

Drug	Targets	Cancer types	Clinical trial ID	Drug status
Pan-FGFR inhibitors				
Rogaratinib	FGFR1–4	Metastatic urothelial carcinoma	NCT03410693	Phase II/III clinical trial
LY2874455	FGFR1–4	Urothelial carcinoma	NCT02529553	Phase I clinical trial
KIN-3248	FGFR1-4	Intrahepatic cholangiocarcinoma, gastric and urothelial carcinoma	NCT05242822	Phase I clinical trial
Erdafitinib	FGFR1–4	bladder cancer	–	Marketed
Futibatinib	FGFR1–4	Cholangiocarcinoma	–	Marketed
Roblitinib	FGFR 1–4	Hepatocellular carcinoma	NCT02325739	Phase I/II clinical trial
Ponatinib	FGFR1–4	Philadelphia chromosome-positive acute lymphoblastic leukemia, chronic myeloid leukemia	–	Marketed (Multi-target kinase inhibitor)
FGFR1/2/3 inhibitors				
Infigratinib	FGFR1–3	Cholangiocarcinoma	–	Market withdrawal
AZD4547	FGFR1–3	squamous cell lung cancer	NCT02965378	Phase 3 clinical trial
Pemigatinib	FGFR1–3	Cholangiocarcinoma, myeloid/lymphoid neoplasms	–	Marketed
HMPL-453	FGFR1–3	Hepatocellular carcinoma, intrahepatic cholangiocarcinoma	NCT04353375	Phase II clinical trial
FGFR2 inhibitors				
RLY-4008	FGFR2	Cholangiocarcinoma	NCT04526106	Phase I/II clinical trial
FGFR3 inhibitors				
LOXO-435	FGFR3	Urothelial cancer, bladder cancer	NCT05614739	Phase I clinical trial
FGFR4 inhibitors				
FGF401	FGFR4	Hepatocellular carcinoma	NCT02325739	Phase I/II clinical trial
Fisogatinib	FGFR4	Hepatocellular carcinoma, colorectal cancer	NCT02508467	Phase I clinical trial

Despite these encouraging outcomes, the broader therapeutic utility of FGFR-TKIs remains constrained by key challenges. These include the problem of resistance, either primary or acquired, which involves distinct mechanisms (gatekeeper *FGFR* mutations, bypassed signaling pathways, …) and is also highly related to intratumorally heterogeneity (see section 6.1.1), limitations in biomarker-based patient selection strategies (see section 6.1.2), and dose-limiting toxicities (both on-targets and off-targets) with adverse events that pose limitations to dose escalation and long-term treatment tolerability (see section 6.1.3)^146^.

#### 6.1.1 Resistance to FGFR-TKIs: a key challenge to overcome

Resistance to FGFR inhibitors can arise from specific point mutations within the FGFR kinase domain, including gatekeeper, molecular brake, and DFG-latch mutations. These alterations disrupt critical hydrogen bonding networks between the receptor and FGFR-TKIs and may also introduce steric hindrance that impairs inhibitor binding^150^. In addition, acquired resistance mutations that emerge during FGFR-TKIs treatment further contribute to diminished drug efficacy and therapeutic failure. Acquired FGFR2^V564F^ gatekeeper mutations have been identified in patients with intrahepatic cholangiocarcinomas harboring FGFR2–BICC1, FGFR2–OPTN, or FGFR2–ZMYM4 fusions^151^. These *FGFR2* alterations confer resistance to pan-FGFR inhibitors by introducing steric hindrance that impairs inhibitor binding. The FGFR1^V561M^ gatekeeper mutation confers resistance to AZD4547 in squamous cell lung cancer by activating STAT3 signaling and facilitating EMT, thereby promoting a more aggressive tumor phenotype^152^. Similarly, FGFR3^V555M^ is a gatekeeper mutation that has been observed as a mechanism of clinical resistance to erdafitinib in FGFR3^+^ bladder cancer^150^.

Activation of alternative RTKs and downstream signaling pathways, such as the RAS–RAF–ERK and PI3K–AKT–mTOR cascades, represents another mechanism by which cancer cells evade FGFR-TKIs. Notably, RTKs such as EGFR and MET, can functionally compensate for FGFR blockade through both genetic alterations and non-genetic adaptive responses^153^. For instance, in head and neck squamous cell carcinoma, AZD4547-resistant cells exhibited elevated phosphorylated EGFR and downstream AKT signaling, which contributed to sustained proliferation despite FGFR inhibition. Co-treatment with the EGFR inhibitor gefitinib restored drug sensitivity, confirming that EGFR serves as a functional bypass node under FGFR-TKI pressure^9^. Similarly, MET signaling has been implicated as an alternative driver in FGFR-dependent tumors. Activation of GAB1 and ERK through MET sustains cell proliferation and confers resistance to FGFR inhibition, a phenotype that can be overcome by combined FGFR-MET blockade^154^. This convergence highlights the plasticity of RTK networks and the importance of co-targeting upstream or parallel receptors. In addition, activating mutations in downstream effectors such as *KRAS* and *PIK3CA*, as well as loss-of-function mutations in the tumor suppressor *PTEN*, can drive constitutive signaling independent of RTKs, further limiting the efficacy of FGFR inhibition^155^.

Another critical barrier is intratumoral heterogeneity (ITH), an inherent feature of solid tumors that complicates uniform therapeutic targeting. FGFR alternative splicing appears to be dynamically regulated across distinct tumor regions^156^. In gastric cancer, profound spatial heterogeneity has been observed in FGFR2-IIIb expression between biopsies, surgical specimens, and metastatic sites. In one cohort of 163 paired biopsy and surgical samples, concordant FGFR2-IIIb overexpression was observed in only 0.6% of cases, while concordance between primary and metastatic lesions was just 0.7%^157^. Similarly, discordant FGFR2-IIIb expression between primary tumors and lymph-node metastases was noted in 44.4% of cases, including both negative and positive conversions. Consistently, paired analyses revealed that both the frequency of FGFR2-IIIb positivity and the proportion of positive tumor cells were significantly higher in metastatic lesions than in primary tumors (8% *vs*. 3%; 75% *vs*. 47%; *P* < 0.001)^158^, highlighting pronounced spatial heterogeneity and further supporting spatial variability of isoform expression within the same patient.

Clinically, such heterogeneity has direct implications for FGFR2-targeted therapy. In the FIGHT and FORTITUDE trials, bemarituzumab showed greater efficacy in patients with ≥10% FGFR2-IIIb-positive cells by IHC, but the predictive reliability of baseline biopsy was limited when spatial heterogeneity was not considered^159^. Liquid biopsies measuring FGFR2 amplification or splice variants *via* circulating tumor DNA (ctDNA) and cell-free RNA (cfRNA) may broaden sampling yet remain technically constrained in resolving isoform-level heterogeneity^160^. To address these challenges, future strategies should integrate multiregional sampling, spatial transcriptomics, and isoform-specific imaging approaches to capture the full landscape of FGFR splicing heterogeneity. Such tools could refine patient stratification and enable adaptive, isoform-guided treatment strategies.

#### 6.1.2 Challenges in biomarker-guided FGFR therapy

Another critical barrier to overcome is the suboptimal performance of current biomarker detection strategies. Although genomic profiling can identify *FGFR* mutations, fusions, and amplifications, false-negative rates of up to 15% have been reported, largely due to the discordance between DNA-level alterations and functional mRNA or protein expression^147^. Conventional sequencing also fails to capture the dynamic expression and clinical relevance of FGFR splice variants, which are key determinants of drug sensitivity, resistance, and patient outcomes^161^. These shortcomings restrict the predictive value of biomarker testing and complicate patient stratification in clinical trials.

Meanwhile, even when FGFR alterations are accurately detected, the therapeutic exploitation of these biomarkers remains limited by the absence of sufficiently selective inhibitors. For example, *FGFR1* amplification in hormone receptor-positive breast cancer has been linked to resistance to cyclin-dependent kinases4/6 inhibitors, yet the lack of FGFR1-selective inhibitors prevents effective targeting of this vulnerability^162^. Moreover, most currently available FGFR inhibitors are pan-FGFR TKIs, which indiscriminately block FGFR1–4. This broad activity increases the risk of dose-limiting toxicities and fails to address the distinct oncogenic contributions of isoform- or tissue-specific FGFR variants.

#### 6.1.3 Adverse effects of FGFR-inhibitors: another critical challenge

The clinical use of FGFR-TKIs is also constrained by characteristic toxicities, most of which stem from on-target inhibition of physiological FGFR signaling in normal tissues. The FGF23–FGFR1 axis plays a central role in phosphate homeostasis, and disruption of this pathway is considered the primary mechanism underlying hyperphosphatemia, a hallmark adverse event associated with FGFR TKIs. Clinical studies report that serum phosphate elevations occur in over 70% of patients^163^. Management strategies typically involve dose modifications and the use of phosphate binders such as sevelamer or lanthanum carbonate.

In addition to metabolic disturbances, gastrointestinal adverse events, including stomatitis, diarrhea, and xerostomia, are commonly reported with selective- and pan-FGFR TKIs such as pemigatinib and futibatinib^164^. These events are thought to reflect the disruption of FGFR-mediated epithelial integrity and homeostasis. Dermatologic toxicities, including alopecia, nail disorders, and palmar-plantar erythrodysesthesia syndrome, are also prevalent^165^. Treatment-related side effects of FGFR inhibitors are not uncommon. Therefore, active monitoring during treatment is possible to minimize dose reductions and discontinuations and would be beneficial to patients' quality of life and outcomes.

### 6.2 Emerging therapeutics to overcome FGFR-TKIs’ limitations: insights from FGFR-RNA splicing crosstalks

As described previously, alternative splicing of FGFRs strongly influence their pattern of cellular and tissular expression as well as their activity in regulating critical biological processes involved in tumorigenesis and escape from therapies^166^. Therefore, and although this remains largely underexplored, the targeting of FGFR–RNA splicing crosstalks could provide novel insights and solutions to overcome the limitations associated with current FGFR-targeted therapies.

#### 6.2.1 FGFR splice variants as promising predictive biomarkers

Traditional biomarkers, such as *FGFR1* amplification in lung squamous cell carcinoma, have shown limited predictive value for response to FGFR-TKI. For instance, the FGFR1 inhibitor nintedanib failed to outperform chemotherapy in the phase III LUME-Lung-1 trial, underscoring the inadequacy of genomic alterations alone as biomarkers^167^^,^^168^. In contrast, the differential expression patterns of FGFR splice variants underscoring their role in tumor heterogeneity could offer more valuable insights for molecular patient stratification and personalized therapy^169^^,^^170^. As an example, the ratio of FGFR1 isoforms (FGFR1*α* and FGFR1*β*) is closely associated with cancer progression and prognosis, with FGFR1*β* being particularly linked to tumor initiation and poor survival outcomes in breast cancer^171^. In addition, high FGFR1*β* expression (reflected in an increased FGFR1*β*/FGFR1*α* ratio) has been linked to enhanced responsiveness to FGFR inhibitors such as BGJ-398^63^. The presence of specific FGFR2 variants may also indicate heightened sensitivity to particular FGFR inhibitors^172^. Consistently, in endometrial cancer, research utilizing patient-derived xenograft (PDX) and organoid (PDXO) models has demonstrated that tumors with high FGFR2-IIIc expression exhibit heightened sensitivity to FGFR inhibitors such as BGJ398 and pemigatinib. These inhibitors also reshape the tumor microenvironment by reducing angiogenesis and M2 macrophage infiltration (CD206^+^). Furthermore, knockdown experiments using FGFR2 shRNA confirmed tumor dependency on FGFR2-IIIc, reinforcing its potential as a precision oncology target^173^. Additionally, recent findings highlight subtype-specific expression of FGFR2 splice variants in breast cancer. The IIIb variant is predominantly expressed in estrogen receptor-positive (ER^+^) breast cancer, whereas the IIIc variant is significantly elevated in HER2-positive breast cancer^174^. These findings suggest FGFR2-IIIc as a candidate biomarker of aggressiveness with potential to guide therapeutic decision-making, particularly in metastatic and recurrent disease. Continued research into the mechanistic roles of FGFR splice variants will be critical to expand their clinical utility as precision biomarkers.

Notably, recent study using Artificial Intelligence (AI)-driven histopathology of H&E slides would offer a cost–effective strategy to infer *FGFR* mutations and splicing alterations by recognizing subtle tumor and stromal patterns^175^^,^^176^. Coupled with multi-omics integration, these platforms could refine biomarker discovery and patient stratification, thereby advancing personalized FGFR-targeted therapies, especially in heterogeneous tumors such as lung squamous cell carcinoma.

#### 6.2.2 Isoform-selective FGFR radiotheranostics for precision diagnosis and treatment

Molecular imaging technologies, particularly positron emission tomography (PET) and single-photon emission computed tomography (SPECT), have emerged as transformative tools for the non-invasive visualization of FGFR expression in tumors. Among these, the FGFR1-targeting probe [^68^Ga]Ga-DOTA-FGFR1 has recently demonstrated significant translational value^177^. In that clinical study, [^68^Ga]Ga-DOTA-FGFR1 enabled clear visualization of both primary and metastatic lung tumors, with higher specificity than [^18^F]FDG for lymph node involvement, and a strong correlation with FGFR1 protein expression (Spearman *r* = 0.6901, *P* < 0.0001). Meanwhile, dosimetry assessments with [^68^Ga]Ga-DOTA-FGFR1-peptide also indicated a favorable safety profile in humans, supporting its potential for routine clinical use^178^. Besides [^68^Ga], a novel PET imaging probe, ^18^F-FGFR1, was recently developed by conjugating a high-affinity FGFR1-targeting peptide with ^18^F-radiolabels and polyethylene glycol (PEG) modifications to improve *in vivo* pharmacokinetics. In that preclinical tumor models with high FGFR1 expression, ^18^F-FGFR1 exhibited excellent stability, specificity, and contrast, enabling effective visualization of FGFR1-expressing lesions with minimal off-target accumulation^179^.

In parallel, the development of FGFR2-targeting SPECT probes has further broadened the imaging repertoire. A series of ^99m^Tc-labeled peptides, particularly [^99^ᵐTc]Tc-FGFR2-1, exhibited specific binding to FGFR2 with excellent tumor uptake and retention in DU145 xenograft models. This tracer achieved significant signal reduction upon co-injection of an FGFR2 competitor CHO2, confirming its specificity and suggesting feasibility for human imaging of FGFR2-expressing tumors^180^.

Extending this strategy to selectively image FGFR splice variants could further enhance diagnostic precision and pave the way for isoform-directed radionuclide therapies. Isoform-specific radiotracers could distinguish epithelial FGFR2-IIIb from mesenchymal FGFR2-IIIc isoforms, or identify truncated decoy receptors, thereby refining tumor classification, revealing spatial heterogeneity, and guiding therapeutic decisions.

Importantly, beyond imaging, coupling therapeutic radionuclides to splice variant-selective ligands represent a promising strategy to directly eliminate tumor cells. This rationale is particularly compelling for FGFR-IIIc, which is consistently upregulated in multiple solid tumors and strongly associated with EMT and metastatic spread. Targeting FGFR-IIIc with radiotheranostics could thus enable selective ablation of disseminated, treatment-resistant tumor populations. Looking forward, multi-modal diagnostic-therapeutic platforms that integrate PET imaging, spatial transcriptomic/proteomic profiling, and isoform-specific radiopharmaceuticals are likely to be transformative in dissecting splicing-driven oncogenesis and overcoming resistance. Such approaches would not only clarify the dynamic role of FGFR isoforms under therapeutic pressure, but also open powerful new avenues for precision oncology interventions against metastatic disease.

#### 6.2.3 Design of novel combinatory therapeutic strategies based on small-molecule inhibitors targeting splicing factors and regulatory kinases

Combination strategies are also a promising approach to mitigate therapeutic resistance to FGFR-TKIs. It has been shown that co-administration of FGFR-TKI with inhibitors targeting compensatory signaling pathways-such as MEK, PI3K, or immune checkpoints can improve response rates and delay resistance. For instance, combining FGFR-TKI with MEK inhibitors yielded an objective response rate of 48% in early clinical studies^181^. Similarly, the combination of lenvatinib (a multikinase inhibitor with anti-FGFR activity) and PD-1 blockade extended progression-free survival (PFS) to 23.9 months in patients with advanced liver cancer^182^. These approaches highlight the rationale of combinatory strategies based on small-molecule inhibitors to overcome resistance and the need to identify new therapeutic combinations.

Owing to the critical role of pre-mRNA splicing deregulation in tumors, small-molecule inhibitors targeting various components of the spliceosome machinery have been developed and appear as promising anti-cancer agents in different cancer types (Table 4 183, 184, 185, 186, 187, 188, 189, 190, 191, 192, 193, 194, 195, 196, 197, 198, 199, 200, 201, 202, 203, 204, 205). More particularly, these compounds target: (1) SF3B1, a core component of the U2 snRNP complex essential for branch point recognition during spliceosome assembly and frequently mutated in hematologic malignancies^206^; (2) SRPK1 and SRPK2 kinases that phosphorylate SR proteins, modulating their nuclear localization and splicing activity; (3) CLK (CDC-like kinases) and DYRK1A/B (Dual-specificity tyrosine-regulated kinases) that also phosphorylate SR proteins and other splicing regulators, linking splicing control to cell cycle and stress responses^207^^,^^208^; (4) Protein arginine methyltransferase that modulate the methylation of splicing factors and RNA-binding proteins, thereby affecting splice site selection and RNA metabolism^168^ (Table 4 183, 184, 185, 186, 187, 188, 189, 190, 191, 192, 193, 194, 195, 196, 197, 198, 199, 200, 201, 202, 203, 204, 205). Early-phase clinical trials of spliceosome inhibitors (*e*.*g*., E7107, H3B-8800) in hematologic malignancies have demonstrated feasibility^209^^,^^210^. In addition, E7107 was shown to preferentially eliminate malignant cells harboring splicing factor mutations (*e*.*g*., *SF3B1*)^211^.

**Table 4:** Emerging therapeutic compounds targeting key regulators of RNA splicing: kinases, methyltransferases, and core spliceosome components.

Target	Cancer	Molecules	Ref.
SF3B1	Lymphoma	Pladienolide B	183
	Acute myeloid leukemia (AML)	H3B-8800	184
		UM4118	185
	Chronic lymphocytic leukemia	Spliceostatin A	186,187
	Chronic lymphocytic leukemia	E7107	188
	Chronic myeloid leukemia	PROTAC-O4I2	189
SRPK1	Cholangiocarcinoma	SPHINX31	190
SRPK1, SRPK2	Melanoma, breast cancer, cholangiocarcinoma	SRPIN340	191, 192, 193
U4/U5/U6 spliceosome	Liver cancer	Isoginkgetin	194,195
CLK1-4, DYRK1A, DYRK1B	Breast cancer	T025	196,197
CLK1, CLK2, CLK4, DYRK1A, DYRK1B, DYRK2	Acute myelogenous leukemia (AML)	BH-30236	198
CLK2	Triple-negative breast cancer	CC-671	199
CLK1, CLK2, CLK4	Melanoma, glioblastoma	SGC-CLK-1	200
CLK2, CLK3	Gastrointestinal cancer	Cirtuvivint	201
PRMT1, PRMT3, PRMT4, PRMT6, PRMT8	Renal cancer	MS023	202
PRMT5	Glioblastoma, breast cancer	GSK591	203, 204, 205

Although this has not been clearly elucidated yet, it is therefore plausible that some of these compounds modulate the pattern of expression of FGFR splice variants in cancer cells, thereby influencing sensitivity to FGFR-TKIs. Hence, SRSF1 which is phosphorylated by SRPKs and CLKs, is a key regulator of FGFR isoforms switching^212^. This raises the hypothesis that co-targeting splicing pathways could suppress the emergence of drug-resistant FGFR splice variants and leverage synthetic lethality in tumors dependent on aberrant splicing for survival. Future investigations are warranted to explore spliceosome inhibitors either as monotherapies or in rational combinations with FGFR inhibitors (isoform-specific or pan-FGFR) in solid tumors driven by FGFR dysregulation.

Moreover, advanced drug delivery platforms, such as liposomes, nanoparticles, and tumor microenvironment-responsive carriers, have been shown to enhance tumor-specific drug accumulation, improve pharmacokinetics, and reduce systemic toxicity^213^. Such delivery systems could be particularly valuable when combined with splicing-targeted therapies to achieve synergistic and more durable anti-tumor effects.

#### 6.2.4 Therapeutical potential of soluble FGFRs in cancer treatment

Soluble Fibroblast Growth Factor Receptors (sFGFRs) are alternatively spliced isoforms lacking the transmembrane and intracellular domains, resulting in secreted extracellular fragments capable of sequestering FGFs and attenuating aberrant FGF/FGFR signaling. Both naturally occurring and bioengineered sFGFRs typically function as decoy receptors, competitively binding FGFs to suppress ligand-induced receptor activation in tumor cells. However, certain sFGFRs may exhibit context-dependent pro-tumorigenic effects, underscoring the complexity of their biological roles^214^^,^^215^.

One of the most clinically advanced examples of FGFR decoy receptor is FP-1039, a recombinant sFGFR1-IIIc fusion protein that binds FGFs with high affinity, disrupting autocrine and paracrine growth loops. Preclinical studies demonstrated its efficacy in inhibiting angiogenesis and tumor cell proliferation, particularly in FGFR1-amplified lung cancers and FGFR2-mutant endometrial cancers^207^^,^^216^. In a Phase Ib clinical trial (NCT01868022), co-administration of FP-1039 with pemetrexed and cisplatin resulted in sustained tumor suppression, supporting its potential role in combination regimens for FGFR-driven malignancies^217^.

Another promising molecule is msFGFR2-IIIc, a soluble FGFR2-IIIc variant harboring the Ser252Trp (S252W) mutation. This engineered receptor fragment potently inhibits FGFR1-IIIc signaling in NCI-H1299 lung cancer cells by preventing FGF2-induced receptor activation. In xenograft models, msFGFR2-IIIc significantly suppressed tumor growth, suggesting its utility in cancers co-expressing FGFR2-IIIc and FGFR1-IIIc isoforms^218^.

Recifercept, a recombinant soluble FGFR3 decoy protein originally developed for achondroplasia, functions by binding FGF ligands and dampening FGFR3-mediated signaling. Despite promising preclinical activity, its clinical development was discontinued due to insufficient efficacy in its original indication^219^. Nevertheless, given the frequent overexpression or mutation of FGFR3 in multiple cancers, repurposing Recifercept or optimizing next-generation FGFR3 decoys may hold therapeutic value in oncology.

Together, these findings highlight the therapeutic potential of sFGFRs, particularly in ligand-dependent FGFR-addicted tumors, includingsubsets with *FGFR* amplification or activating mutations. Future efforts should focus on optimizing sFGFR pharmacokinetics, improving isoform selectivity, and evaluating their role in rational combination therapies within the framework of precision oncology.

#### 6.2.5 Antisense oligonucleotides (ASOs) to correct aberrant FGFR splicing

ASOs are short, chemically modified nucleic acid sequences designed to hybridize with target RNAs in a sequence-specific manner. Their mechanisms of action include RNase H1-mediated degradation of mRNA, modulation of alternative splicing, and translational repression *via* interference with ribosomal assembly^220^^,^^221^. In the context of FGFR biology, ASOs provide a powerful platform for selectively correcting aberrant splicing events that drive oncogenic isoform expression.

Targeting splicing regulatory elements within *FGFR* pre-mRNA has yielded promising results. For instance, Bruno et al.^71^ designed ASOs that bind an intronic splicing silencer (ISS) element within FGFR1, resulting in enhanced exon inclusion and restoration of functional receptor isoforms in glioblastoma cells. This strategy increased exon incorporation from 10% to over 70%, underscoring the feasibility of splicing modulation as a therapeutic intervention.

ASO-based strategies offer several advantages for precision targeting of FGFR splice variants. By promoting inclusion or skipping of exons encoding ligand-binding or dimerization domains, ASOs can modulate isoform ratios, restore receptor functionality, or induce nonsense-mediated decay of pathogenic transcripts. These approaches are particularly valuable for highly selective and specific targeting oncogenic FGFR1–3 splice variants that are otherwise difficult to inhibit with small molecule kinase inhibitors.

In addition to ASOs, small molecule splicing modulators offer complementary strategies. Risdiplam, an FDA-approved small molecule for spinal muscular atrophy, exemplifies the translational potential of splicing-targeted therapy by promoting exon inclusion to restore protein function^222^. Such agents could be adapted to modulate FGFR splicing in cancer and other diseases characterized by aberrant splicing.

Given the high prevalence of splicing dysregulation in malignancies, including recurrent mutations in splicing factors and misexpression of FGFR isoforms, splicing correction strategies represent a frontier in targeted cancer therapy^49^^,^^223^. Developing isoform specific ASOs as well as small molecule modulators will facilitate the integration of RNA-based therapeutics into precision oncology pipelines, offering new avenues to overcome resistance and improve clinical outcomes in FGFR-driven tumors^224^.

#### 6.2.6 Additional emerging strategies on targeting FGFR and its splice variants

The advent of “next-generation” FGFR-targeted agents offers new opportunities to address isoform-specific resistance and reduce toxicity. These include monoclonal antibodies, PROTACs (proteolysis-targeting chimeras), RNA/DNA aptamers, mini-proteins, or cell-based therapies^10^^,^^222^. Some of these approaches can enable selective blockade of pathogenic FGFR splice variants while sparing physiological isoforms, thereby minimizing on-target adverse effects. A collection of emerging biological agents specifically targeting FGFRs as well as their splice variants with their stage of development is provided in Table 5 ^112^^,^^217^^,^^225^, ^226^, ^227^, ^228^, ^229^, ^230^, ^231^, ^232^, ^233^, ^234^, ^235^, ^236^, ^237^, ^238^, ^239^, ^240^, ^241^, ^242^, ^243^.

**Table 5:** Emerging biological- and cell-based therapies targeting FGFRs and their splicing isoforms.

Receptor	Drug	Mechanism of action	Stage of development	Clinical trial	Ref.
FGFR1	IMC-A1	Anti-FGFR1-IIIc mAb	Preclinical	–	225
	FP-1039 (GSK3052230)	Soluble FGFR1-IIIc trapping mitogenic FGFs	Clinical trial	NCT00687505 NCT01868022	217,226
	LG1188	AZD4547-based PROTAC	Preclinical	–	227
	VZ23	Anti-FGFR1-IIIb/c DNA aptamer	Preclinical	–	228
	S2h	AZD4547-based PROTAC	Preclinical	–	229
FGFR1/2	DGY-09-192	Infigratinib-based PROTAC	Preclinical	–	230
FGFR2	Aprutumab	Anti-FGFR2 mAb	Clinical trial	NCT01881217	231
	LC-MB12	Infigratinib-based PROTAC	Preclinical	–	232
	N5	Erdafitinib-based PROTAC	Preclinical	–	233
	Anti-FGFR2- (IIIc) research antibody	Anti-FGFR2-IIIc mAb	Preclinical	–	234
	GP369	Anti-FGFR2-IIIb mAb	Preclinical	–	112
	Bemarituzumab (FPA144)	Anti-FGFR2-IIIb mAb	Clinical trial	NCT05052801 NCT05111626	235,236
	Apt-46	Anti-FGFR2-IIIb DNA aptamer	Preclinical	–	237
	LC-MB12	Proteolysis-targeting chimeras	Preclinical	–	232
	3H-3000	Anti-FGFR2-IIIb mAb	Preclinical	–	238
FGFR3	Recifercept	Soluble FGFR3-IIIc, trapping FGF1, FGF9, FGF16 & others	Clinical trial	NCT04638153	239
	LY3076226	Anti-FGFR3 ADC	Clinical trial	NCT02529553	240
	iR3	Anti-FGFR3 RNA aptamer	Preclinical	–	241
FGFR4	RJ150HL and RJ154HL	FGFR4-targeting CAR-T cells	Preclinical	–	242
	U3-1784	Anti-FGFR4 mAb	Clinical trial	NCT02690350	243

##### 6.2.6.1 Monoclonal antibodies

Several monoclonal antibodies have been developed to selectively target FGFR2 splice variants, particularly FGFR2-IIIb. Bemarituzumab is a monoclonal antibody that specifically targets FGFR2-IIIb, blocking its interaction with canonical ligands such as FGF7, FGF10, and FGF22. This inhibition disrupts ligand-dependent FGFR2-IIIb signaling and downstream oncogenic pathways. Bemarituzumab also features an engineered Fc domain that enhances antibody-dependent cellular cytotoxicity (ADCC), facilitating immune-mediated tumor clearance through natural killer (NK) cells^244^.

Aprutumab is a fully humanized antibody recognizing conserved residues (P23, L27, E29) in the N-terminal domain shared by FGFR2-IIIb and FGFR2-IIIc. It induces receptor internalization and lysosomal degradation *via* RAB7-positive endosomes in FGFR2-expressing cell lines such as SUM-52PE and SNU16^245^. Aprutumab is also under evaluation as a radioimmunoconjugate platform, expanding its potential in targeted radionuclide therapy^246^. 3H-3000 is a humanized IgG1κ monoclonal antibody with high specificity for FGFR2-IIIb. It potently suppresses FGF7-induced receptor phosphorylation and proliferation in FGFR2-IIIb-overexpressing gastric cancer cells (*e*.*g*., SNU16). Engineering modifications have enhanced its ADCC function. In xenograft models, 3H-3000 significantly inhibits tumor growth, underscoring its therapeutic promise for FGFR2-IIIb-driven tumors^238^.

##### 6.2.6.2 Proteolysis-targeting chimeras (PROTACs)

PROTACs represent an innovative class of therapeutic agents designed to selectively degrade pathogenic proteins through hijacking the ubiquitin-proteasome system. These bifunctional molecules simultaneously bind to the target proteins and E3 ubiquitin ligases, leading to its proteasomal degradation^247^. Compared to traditional small-molecule inhibitors, PROTACs offer the advantage of delivering more potent and sustained inhibitory effects^248^. Recent advances in the development of FGFR-targeting PROTACs have significantly broadened the therapeutic landscape by achieving improved selectivity towards different FGFRs and enhanced antitumor efficacy. A representative example is compound S2h, developed by Wang et al.^229^, which induces potent and selective degradation of FGFR1 with a DC_50_ of 39.78 nmol/L and a maximal degradation rate of 78% in FGFR1-overexpressing KG1a cells, while exhibiting negligible activity against FGFR2-4. Mechanistically, S2h functions through a cereblon (CRBN)-dependent, ubiquitin–proteasome-mediated degradation pathway. LC-MB12, an Infigratinib-based PROTAC, selectively degrades FGFR2 and effectively inhibits the proliferation of TEL–FGFR2 fusion-positive Ba/F3 cells and FGFR2-overexpressing gastric cancer cells (SNU16)^232^. LC-MB12 induces asymmetric FGFR2 dimer disruption and disassembly of the FRS2/SHP2/GRB2 complex, effectively dismantling scaffold-based signal propagation^232^. Likewise, the FGFR2-specific degrader N5, generated by coupling erdafitinib to a CRBN ligand, achieves exceptional FGFR2 degradation (DC_50_ = 0.03 nmol/L) with minimal off-target activity, and exerts strong antiproliferative effects in FGFR2-amplified gastric cancer cells and xenografts^233^. Similarly, DGY-09-192, another FGFR-targeting PROTAC also derived from Infigratinib, utilizes the von Hippel–Lindau (VHL) E3 ligase to selectively degrade FGFR1 and FGFR2^122^. Additionally, DGY-09-192 degraded mutant FGFR1/2, blocking mutant receptor-induced signaling and antiestrogen resistance^249^. In ER-positive breast cancer, co-treatment with DGY-09-192 and the ER*α* degrader fulvestrant led to complete inhibition of cell proliferation and significant tumor regression in ER-positive, FGFR1-amplified patient-derived xenograft models. LG1188, another representative PROTAC, functions as a selective and efficient FGFR1 degrader with potential therapeutic relevance^227^

Although these agents remain in preclinical development, they highlight the potential of targeted protein degradation as an alternative to conventional kinase inhibition, particularly in FGFR-driven malignancies exhibiting resistance or limited response to current therapies. Importantly, in the context of FGFR splice variants, the next frontier lies in engineering PROTACs capable of selectively degrading oncogenic FGFR splice isoforms, thereby achieving greater therapeutic specificity and minimizing on- and off-target toxicity.

##### 6.2.6.3 DNA and RNA aptamers

Nucleic acid aptamers represent a versatile and highly specific class of oligonucleotide-based therapeutics capable of modulating protein function with antibody-like affinity and selectivity.

VZ23, a DNA aptamer, has been shown to bind both FGFR1-IIIb and FGFR1-IIIc isoforms, effectively blocking downstream signaling cascades and inhibiting FGFR1-mediated cellular processes. Notably, VZ23 does not exhibit cross-reactivity with FGFR2, FGFR3, or FGFR4, highlighting its potential as a highly selective therapeutic for FGFR1-driven malignancies^250^. Similarly, Apt-46, a newly synthesized DNA aptamer, targets FGFR2-IIIb and inhibits its activation in cancer cells^237^. Beyond DNA aptamers, RNA aptamers such as iR3 (anti-FGFR3) and RBM-007 (anti-FGF2) have shown efficacy in preclinical and clinical studies for various conditions, including age-related macular degeneration (AMD) and lung cancer^241^^,^^251^. These RNA aptamers disrupt ligand–receptor interactions or dimerization, thereby attenuating aberrant FGFR signaling. The high specificity, low immunogenicity, and modularity of aptamers also support their potential application in selectively targeting oncogenic FGFR splice variants.

##### 6.2.6.4 Mini-proteins for isoform-specific FGFR targeting

Monoclonal antibodies targeting RTKs may encounter limitations in epitope accessibility and binding efficiency, particularly when targeting splice variants with small or conformationally restricted extracellular domains. Engineered mini-proteins represent a promising alternative, offering high-affinity, isoform-specific binding with reduced steric hindrance. The computationally designed mini-protein mb7 selectively binds the D3 domain of FGFR1–3 IIIc isoforms, effectively competing with natural ligands and Klotho co-receptors to block FGFRc-mediated signaling^252^. This targeted approach enables selective inhibition of oncogenic FGFR IIIc pathways while sparing FGFR IIIb isoforms, thereby enhancing therapeutic specificity.

Preclinical studies have demonstrated that mb7 attenuates FGFRc-driven cellular responses and exhibits anti-angiogenic activity in tumor models^253^. Given their favorable pharmacological properties, mini-proteins like mb7 offer a versatile platform for isoform-specific inhibition, with potential applications in solid tumors, regenerative medicine, and angiogenesis-associated pathologies. Ongoing optimization of binding affinity, stability, and *in vivo* delivery systems will again be essential to advance their clinical translation.

##### 6.2.6.5 Chimeric antigen receptor (CAR) T-cell therapy

The advent of biologics and chimeric antigen receptor (CAR) T-cell therapies has opened new avenues for the selective targeting of FGFR family members, with the conceptual possibility of discriminating between FGFR splice isoforms including FGFR1-IIIb/IIIc, FGFR2-IIIb/IIIc, and FGFR3-IIIb/IIIc. CAR T-cell therapy has emerged as a transformative modality for hematologic malignancies and is now being explored in solid tumors with aberrant FGFR signaling. In pediatric rhabdomyosarcoma, for instance, FGFR4 is frequently overexpressed under the influence of PAX3–FOXO1 fusion oncogenes. While small-molecule FGFR inhibitors show limited efficacy due to resistance-conferring mutations (*e*.*g*., N535K, V550L), FGFR4-directed CAR-T cells have been developed to overcome this barrier through immune-mediated cytotoxicity^254^. Two preclinical CAR-T constructs, RJ154-HL and 3A11, utilizing distinct single-chain variable fragments (scFvs) specific for FGFR4, have demonstrated potent anti-tumor activity. In orthotopic xenograft models of rhabdomyosarcoma, RJ154-HL CAR-T cells effectively suppressed tumor growth, especially when combined with agents targeting the myeloid compartment of the tumor microenvironment^242^^,^^255^.

Designing CAR constructs that recognize isoform-specific extracellular epitopes, particularly in regions encoded by alternative exons (*e*.*g*., exon 8 *vs*. exon 9 in FGFR2), offers a strategy to achieve precise elimination of malignant cells expressing oncogenic variants, while sparing normal tissues that express physiological isoforms. Incorporating splicing-informed antigen design and advanced CAR architectures, such as logic-gated or dual-antigen systems, holds promise for enhancing therapeutic precision, efficacy, and safety in FGFR-driven cancers.

## 7 Trends and future research directions

### 7.1 Deciphering FGFR splicing complexity through single-cell and spatiotemporal transcriptomics

Single-cell RNA sequencing (scRNA-seq) has enabled unprecedented resolution in characterizing cellular heterogeneity and transcriptomic complexity within tumors. While traditionally applied to quantify gene expression, emerging adaptations of scRNA-seq now allow to investigate alternative splicing including that of FGFR^256^^,^^257^. Historically, limitations in sequencing depth and computational algorithms restricted splicing analyses at the single-cell level^258^. However, recent innovations, including Chigene scRNA-seq, Molecular crowding Single-Cell RNA Barcoding and Sequencing, and Smart-seq2, have overcome many of these constraints by enhancing cDNA synthesis efficiency, reverse transcription fidelity, and isoform detection sensitivity^259^, ^260^, ^261^.

In oncological contexts, scRNA-seq offers a powerful approach to resolve FGFR splice-variant expression within heterogeneous tumor ecosystems, including lung, breast, and bladder cancers. A key example is FGFR2 isoform switching during EMT which displays distinct cell-type-specific expression patterns and highlights the added value of single-cell resolution^256^^,^^262^. Using Smart-seq2, investigators further identified ESRP1/2 as critical regulators of FGFR2 splicing during EMT, thereby providing mechanistic insight into how splicing contributes to tumor plasticity^263^. Further advancing this field, GoT-Splice, which is a multimodal single-cell platform, integrates transcriptome, surface proteome, somatic mutation, and splicing data to provide a holistic view of regulatory events at the single-cell level^264^. Although its application in FGFR field remains nascent, GoT-Splice has successfully mapped SF3B1 mutation-associated splicing disruptions in myelodysplastic syndromes.

Complementing scRNA-seq, spatial transcriptomics (ST) offers spatial context to gene expression within intact tissue sections, preserving microenvironmental architecture. By integrating ST with single-cell technologies, researchers can resolve the spatial distribution of FGFR splice isoforms within tumors, elucidating regional heterogeneity and niche-specific signaling cues^265^^,^^266^. For instance, in hepatocellular carcinoma, differential expression of FGFR variants between tumor cores and invasive edges has been linked to metastatic potential and angiogenic profiles^267^.

Together, the integration of scRNA-seq and ST provides a multidimensional framework for dissecting FGFR splicing events at both high resolution and spatial precision^268^. Such approaches will be invaluable for identifying isoform-specific therapeutic vulnerabilities, guiding spatially targeted interventions.

### 7.2 CRISPR/Cas9-mediated regulation of FGFR splicing: opportunities and challenges

CRISPR/Cas9-based genome editing has emerged as a powerful tool for interrogating and manipulating alternative splicing at unprecedented specificity^269^. By designing guide RNAs to target intronic or exonic splice regulatory elements within *FGFR* loci, researchers can modulate exon inclusion or skipping, thereby engineering splice isoforms associated with oncogenic signaling or drug resistance^270^^,^^271^. Such targeted editing has elucidated the role of splicing in resistance to tyrosine kinase inhibitors (TKIs) and enabled the creation of isogenic models for testing isoform-specific inhibitors.

CRISPR/Cas9 has been utilized to generate targeted knockout models of SR proteins and their isoforms^272^, enabling functional interrogation of their roles in FGFR splicing. In parallel, CRISPR-based screens have identified regulators of FGFR, such as ILK, which modulates FGFR inhibitor sensitivity in gastric cancer^273^. Similarly, CRISPR/Cas9-mediated knockout of *Ninj2*, a modulator of multiple RTKs including FGFR, demonstrated suppression of glioma cell survival and invasiveness, reinforcing broader intersection between RTK signaling and FGFR pathway regulation^274^.

However, translating CRISPR-based splicing modulation into clinical applications entails several critical challenges. One major concern is the off-target effect, which may cause unintended DNA cleavage at genomic loci with partial homology to the guide RNA^275^. Studies have shown that Cas9 can tolerate up to 3–5 mismatches in single-stranded guided RNA (sgRNA) recognition, raising the risk of non-specific double-strand breaks (DSBs), genomic instability, or unintended oncogenic activation^276^. Moreover, achieving effective delivery of CRISPR components into tumor tissues, especially in solid malignancies, is hindered by biological barriers such as stromal density, endosomal trapping, and systemic degradation^277^. Immunogenicity is another important consideration: the Cas9 protein is of bacterial origin, and pre-existing immunity or innate immune activation may compromise therapeutic efficacy and safety^278^.

To address these limitations, several technological advancements have emerged. High-fidelity Cas9 variants, including SpCas9-HF1 and eSpCas9, introduce rational mutations to minimize off-target DNA binding while preserving editing efficiency^279^. Cas9 nickase (nCas9) and base editors represent additional refinements: the former induces single-strand nicks rather than DSBs, while the latter enables precise nucleotide changes without cleaving the DNA backbone, substantially reducing genotoxicity^276^. On the delivery front, non-viral carriers such as lipid nanoparticles (LNPs) and biodegradable polymeric systems have shown promise in encapsulating *Cas9* mRNA or ribonucleoprotein (RNP) complexes for tissue-specific targeting, with improved endosomal escape and reduced systemic toxicity^278^^,^^280^^,^^281^.

### 7.3 Functional and therapeutic roles of lncRNAs in FGFR alternative splicing

LncRNAs are transcripts longer than 200 nucleotides that lack protein-coding potential^282^. Once considered transcriptional noise, they are now recognized as versatile regulators of gene expression, influencing alternative splicing, translation, chromatin remodeling, nuclear organization, and epigenetic modifications^283^^,^^284^. In cancer, aberrant lncRNA expression contributes to tumor initiation, progression, and therapeutic resistance^285^. Within this context, growing evidence implicates lncRNAs as important modulators of FGFR alternative splicing. Mechanistically, lncRNAs regulate exon recognition and spliceosome assembly through RNA–protein and RNA–chromatin interactions^286^. Likely in hepatocellular carcinoma, specific lncRNAs recruit polycomb proteins (EZH2, SUZ12) and histone demethylases (KDM2A) to *FGFR2* loci, facilitating exon IIIb inclusion and favoring tumor-suppressive isoform expression^287^. Additionally, lncRNAs can indirectly regulate FGFR splicing by modulating the expression or activity of splicing factors, such as SRSF1 and hnRNP A1^288^.

Despite their therapeutic promise, lncRNA-based interventions face key challenges, including RNA instability, limited target specificity, and inefficient *in vivo* delivery. To address these issues, advances in nucleic acid chemistry (*e*.*g*., backbone and base modifications) and delivery systems (viral vectors, LNPs, polymer carriers, inorganic nanoparticles, protein-based platforms, and exosomes) are under active investigation^289^^,^^290^. However, neither chemical modifications nor delivery systems alone fully overcome these barriers. Combining optimized nucleic acid modifications with next-generation delivery technologies represents a promising avenue to unlock the therapeutic potential of lncRNA-targeted strategies in FGFR-driven cancers.

**Figure 4:**
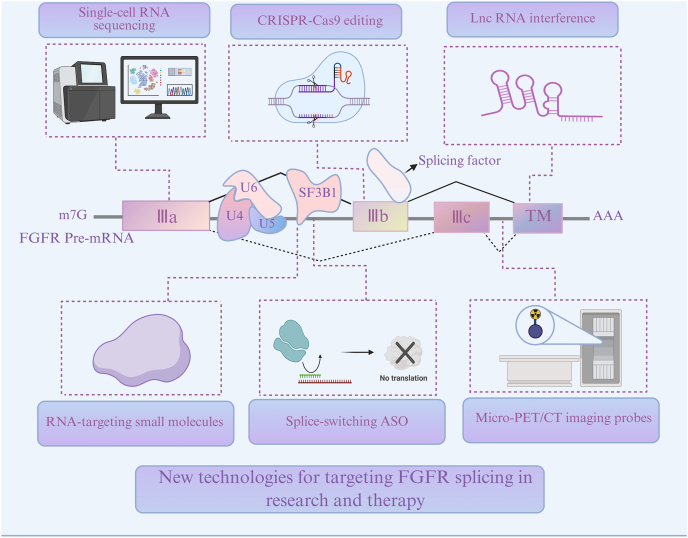
Emerging technologies for deciphering FGFR splicing mechanisms and for developing precise, specific, and selective strategies to therapeutically target FGFR splicing. Figure created with BioRender.

Collectively, the integration of CRISPR/Cas9, lncRNA-based modulation, and single-cell transcriptomics offers a powerful platform to dissect FGFR splicing regulation and develop personalized, isoform-targeted therapeutics in FGFR-driven cancers (Fig. 4).

## 8 Conclusions

To the best of our knowledge, this work provides the first comprehensive review of the bi-directional interactions between FGFR signaling and RNA splicing, highlighting their critical roles in oncogenesis, tumor progression, and therapeutic resistance. FGFR splice variants have emerged as key regulators of EMT, cancer metastasis and drug resistance, demonstrating their potential as promising biomarkers and therapeutic targets. We have examined how alternative splicing governs FGFR isoform diversity and functions, and conversely, how FGFR activation reshapes splicing programs to drive malignancies. These intricate crosstalks underscore the necessity for highly selective and precise therapeutic strategies.

Although FGFR inhibitors, particularly TKIs, have demonstrated clinical efficacy, their long-term success is limited by resistance mechanisms, on- and off-target side effects, and tumor heterogeneity. Insights gained from FGFR–RNA splicing interactions should provide new opportunities to overcome these limitations by targeting both FGFR signaling and the splicing machinery.

The landscape of FGFR isoform-specific targeted therapies continues to evolve, with cutting-edge modalities such as monoclonal antibodies, PROTACs, aptamers, mini-proteins, and CAR-T cells offering new precision oncology avenues. Each of these approaches presents unique advantages: DNA/RNA aptamers provide high specificity, while CAR-T cells deliver potent cytotoxicity against FGFR-expressing tumor cells. As these novel therapies progress toward clinical translation, their further optimization and integration into existing treatment frameworks will be crucial to improving patient outcomes.

Additionally, advancements in transcriptomics, particularly single-cell full-length transcriptomics combined with spatiotemporal analysis, are revolutionizing FGFR splicing research. The incorporation of AI-driven diagnostic tools and CRISPR/Cas9-based editing for precise FGFR splice variant modulation marks a paradigm shift in personalized oncology. Antisense oligonucleotides (ASOs) and small-molecule splicing modulators offer new therapeutic avenues for targeting oncogenic splice variants, further expanding the arsenal against FGFR (isoforms)-driven cancers.

Future research should prioritize translating these insights into clinically actionable strategies. Key steps include identifying patient subgroups defined by specific FGFR splicing patterns, integrating multi-omics data to refine precision medicine applications, and optimizing therapeutic combinations. Importantly, we propose that targeting FGFR splicing isoforms with radiotheranostic approaches may offer a powerful new avenue to both visualize and eradicate aggressive tumor populations. By integrating isoform-directed radiotheranostics with splicing modulation, it will be possible to overcome current limitations and advance toward highly individualized, durable treatments for FGFR-driven malignancies.

## Author contributions

Xuquan Xian, Ruyi Gong, Shunzi Rong, Zhihao Zhang, and Fengtong Jia collected the literature data and prepared the original draft. Xuquan Xian, Lin Li, Zhengguo Chen, Beatrice Eymin and Tao Jia contributed to manuscript review and revision. Tao Jia conceived the idea, provided overall guidance on the scientific framework, supervised the students, acquired funding, and finalized the manuscript revision.

## Conflicts of interest

The authors have no conflicts of interest to declare.
